# Chemical‐Assembled Macrophage‐Engaging Glypican‐3 × Signal Regulatory Protein‐α (SIRP‐α) Bispecific Antibody for Immunotherapy Against Liver Cancer

**DOI:** 10.1002/mco2.70808

**Published:** 2026-06-17

**Authors:** Bo Tang, Wing Hei Leung, Chihao Shao, Dongfang Li, Kwai Man Lau, Macro K. H. Lui, Jingyi Liu, Jacky C. H. Chu, Clare S. W. Yan, Chi‐Ming Che, Xuechen Li, Wing‐Tak Wong, Larry Ming Cheung Chow, Terence K. W. Lee, Clarence T. T. Wong

**Affiliations:** ^1^ Department of Applied Biology and Chemical Technology The Hong Kong Polytechnic University Kowloon Hong Kong China; ^2^ Department of Chemistry University of Hong Kong Pokfulam Hong Kong China; ^3^ Laboratory for Synthetic Chemistry and Chemical Biology Limited New Territories Hong Kong China; ^4^ PolyU‐BGI Joint Research Centre for Genomics and Synthetic Biology in Global Ocean Resources The Hong Kong Polytechnic University Kowloon Hong Kong China

**Keywords:** bioconjugation, bispecific antibody, immunotherapy, macrophage‐engager

## Abstract

Bispecific antibodies (BsAbs) represent a growing class of cancer immunotherapeutics, yet their wider adoption remains limited by complex recombinant production and limited manufacturing efficiency. Here, we report a chemically assembled macrophage‐engaging glypican‐3 × signal regulatory protein‐α (SIRP‐α) bispecific antibody for the treatment of liver cancer. Using our bifunctional linker, glypican‐3‐binding peptide dimers were conjugated to a native anti‐SIRP‐α IgG via a rapid one‐pot, two‐step process, yielding a novel dendritic bispecific antibody (dBsAb). Such a construct simultaneously targets glypican‐3‐positive hepatocellular carcinoma cells and blocks the CD47–SIRP‐α axis, thereby promoting macrophage engagement and phagocytosis. In vitro studies showed substantially enhanced macrophage adhesion and tumor cell clearance compared with the monoclonal antibody. In an in vivo experiment, dBsAb demonstrated a 68% reduction in tumor growth, with increased macrophage infiltration, M1 polarization, and elevated antigen presentation without observable systemic toxicity. This chemical assembly approach offers a practical alternative to recombinant methods for constructing bispecific antibodies and may facilitate the development of macrophage‐directed immunotherapies.

## Graphical abstract

We developed a novel chemical strategy to generate a glypican‐3 × SIRP‐α bispecific antibody that simultaneously blocks the CD47 “don't eat me” signal, engages macrophages to target liver cancer cells for enhanced phagocytosis, and antigen presentation. This novel combination of targets demonstrates remarkable efficacy in vitro and in vivo. This plug‐and‐play platform offers a rapid, scalable approach to producing novel bispecific antibodies for cancer immunotherapy.

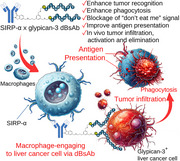

## 1 Introduction

**FIGURE 1:**
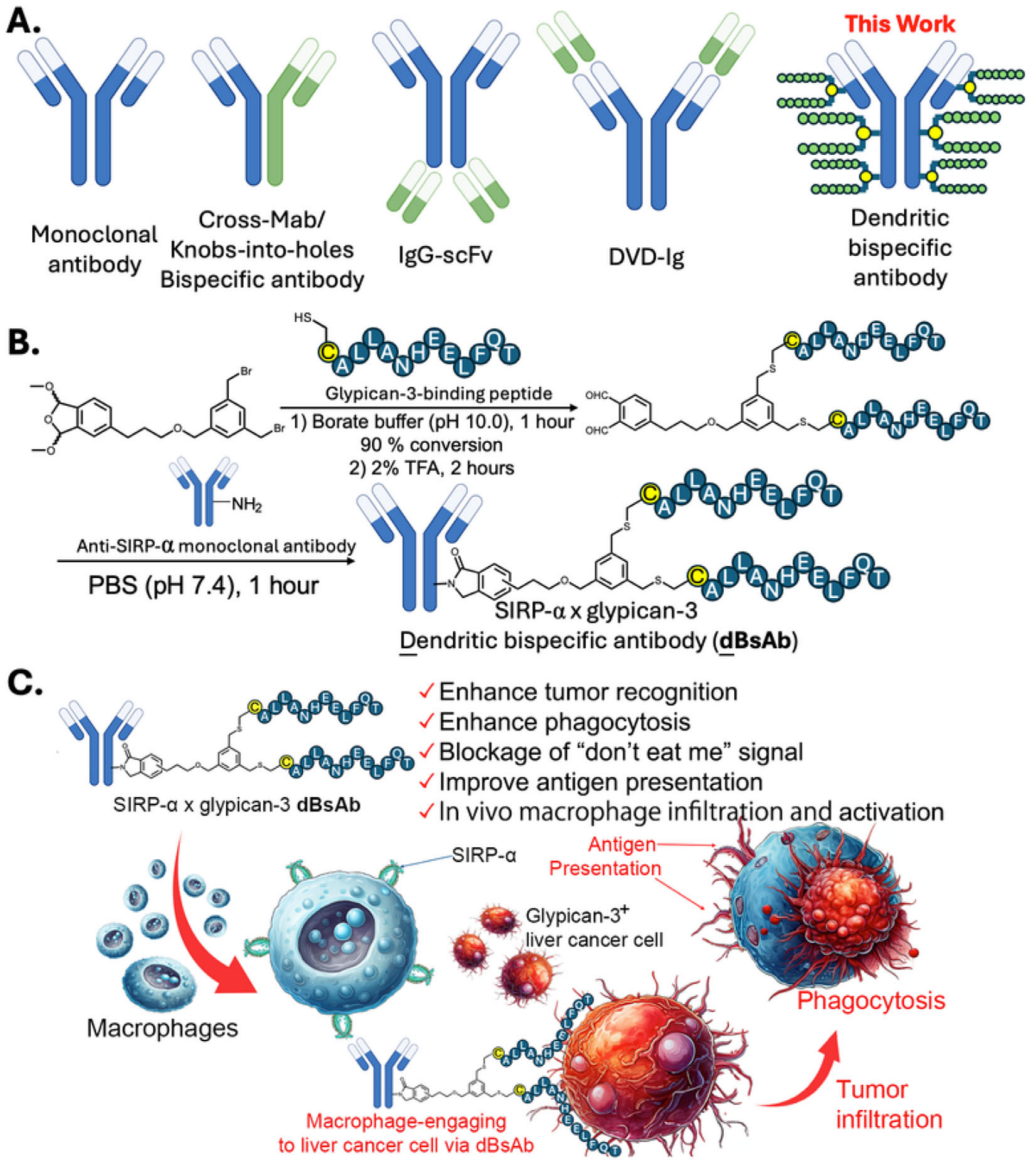
(A) Schematic illustration of different constructs of monoclonal antibodies and bispecific antibodies. (B) Scheme of one‐pot antibody modification with peptide dimers to generate dBsAb. (C) Proposed anticancer mechanism of the novel macrophage‐engaging dBsAb.

Bispecific antibodies (BsAbs) are engineered proteins that can bind to two distinct molecular targets in one entity, which allows simultaneous modulation of two biological targets [1, 2, 3, 4]. Such a mechanism can improve therapeutic outcomes by redirecting immune cells toward tumor cells or by simultaneously modulating multiple signaling pathways [5, 6, 7, 8]. Since their initial development in the 1980s, a wide range of bispecific formats has been explored, such as tandem single‐chain variable fragments (scFvs), dual‐variable‐domain immunoglobulins (DVD‐Ig), and IgG‐like architectures (Figure 1A) [9, 10, 11, 12]. Among these designs, IgG‐based formats are favored for their structural stability, longer serum half‐life, and inducing immune effector functions through the Fc domain [13].

Today, there are only 13 bispecific antibodies that have received global regulatory approval, and more than 300 candidates are in clinical trials, predominantly in oncology [14, 15]. Among approved agents, glofitamab (Columvi), a CD20×CD3 IgG‐like bispecific for relapsed/refractory diffuse large B‐cell lymphoma, achieved an overall response rate (ORR) of 52% with a 39% complete response rate in pivotal trials [14]. Mosunetuzumab (Lunsumio), another CD20×CD3 bispecific approved for follicular lymphoma [16]. Amivantamab (Rybrevant), targeting EGFR and MET in non‐small cell lung cancer, showed a 45% ORR and median duration of response of 7.2 months in heavily pretreated patients [17, 18]. These response rates exceed those reported for monospecific antibodies against the same targets, which often yield ORRs under 30% in comparable settings [15]. Currently, most bispecific antibodies developed to date are focused on T‐cell engagement by linking a tumor‐associated antigen on cancer cells to CD3‐positive cytotoxic T cells. This strategy has proven effective in non‐solid tumors, such as hematological malignancies, due to greater tumor accessibility and lower immunosuppression by the microenvironment. However, T cells have become less effective in solid tumors largely due to the poor T‐cell infiltration, immunosuppressive tumor microenvironment, and safety concerns such as cytokine release syndrome [15].

On the other hand, macrophage‐engaging bispecific antibodies remain rare in the scientific community, and no macrophage‐engaging bispecifics have reached the market despite their considerable potential. Macrophages can infiltrate dense tumor tissue, allowing them to reach areas often inaccessible to T cells. They can directly engulf tumor cells by phagocytosis and initiate broader immune activation through antigen presentation and further stimulate adaptive immune responses.

A major obstacle to BsAb development is the complexity of recombinant production [19]. Traditional methods, such as quadroma technology or knobs‐into‐holes engineering, often suffer from low yields and mispairing of heavy and light chains [20, 21, 22, 23]. All these factors lead to difficulties, from R&D to large‐scale manufacturing and quality control, limiting scalability and increasing production costs. In recent years, chemical approaches to generating bispecifics have gained attention, aiming to offer advantages such as flexible design, rapid synthesis, cost‐effectiveness, and scalability [24, 25]. Doppalapudi et al. reported the first chemical generation of a bispecific antibody by joining two different peptides with a branched azetidinone and fusing them to a scaffold antibody under mild conditions [26]. Later on, more research groups tried to use different bio‐orthogonal or bioconjugation chemistries to generate different scaffolds of bispecific antibodies [27, 28, 29, 30, 31, 32].

In this work, we designed a novel bispecific antibody construct called dendritic bispecific antibody (dBsAb) to investigate the unprecedented bispecific design and target combinations, which also further simplify the design and production of bispecific antibodies. Peptide dendrimers have been widely used in biological applications due to their multivalency and high surface functionality, which enhance binding affinity, avidity, and specificity [33, 34, 35]. We envisage that by conjugating multiple tumor‐targeting peptide dimers on a monoclonal antibody core, forming a dendritic structure could give the antibody an extra tumor‐targeting function with enhanced affinity and avidity (Figure 1). Such a dendrimeric design could also compensate for the weaker binding affinity of single short peptides compared to antibodies. To demonstrate the novel dBsAb, we challenged ourselves to explore an unconventional combination of targets. Among numerous potential targets, we chose to explore the glypican‐3 and SIRP‐α receptors in our first dBsAb design. Glypican‐3 is a recently discovered tumor‐specific antigen that is highly expressed on the liver cancer cell surface, making it an ideal target for tumor recognition [36], while SIRP‐α is involved in immune evasion by inhibiting macrophage activity, and its blockade enhances macrophage‐mediated tumor clearance [37, 38, 39, 40]. Macrophage engagement is advantageous in solid tumors like liver cancer, as macrophages can migrate and infiltrate into the core of solid tumors, making them effective for tumor clearance [41, 42, 43]. Conjugating multiple copies of glypican‐3‐targeting peptide dimer to a SIRP‐α monoclonal antibody confers dual binding capabilities, effectively transforming the molecule into a macrophage‐engaging bispecific antibody (Figure 1B). In theory, the resulting dBsAb addresses three challenges in liver cancer therapy: (i) specifically targeting glypican‐3 for selective tumor targeting, (ii) engaging macrophages and blocking the CD47–SIRP‐α axis to activate macrophages to attack cancer cells, and (iii) enhancing phagocytosis results as well as antigen presentation to further activate other effector cells for more potent immunotherapy (Figure 1C).

## 2 Results

### 2.1 Chemical Generation of Novel Dendritic Bispecific Antibody

**FIGURE 2:**
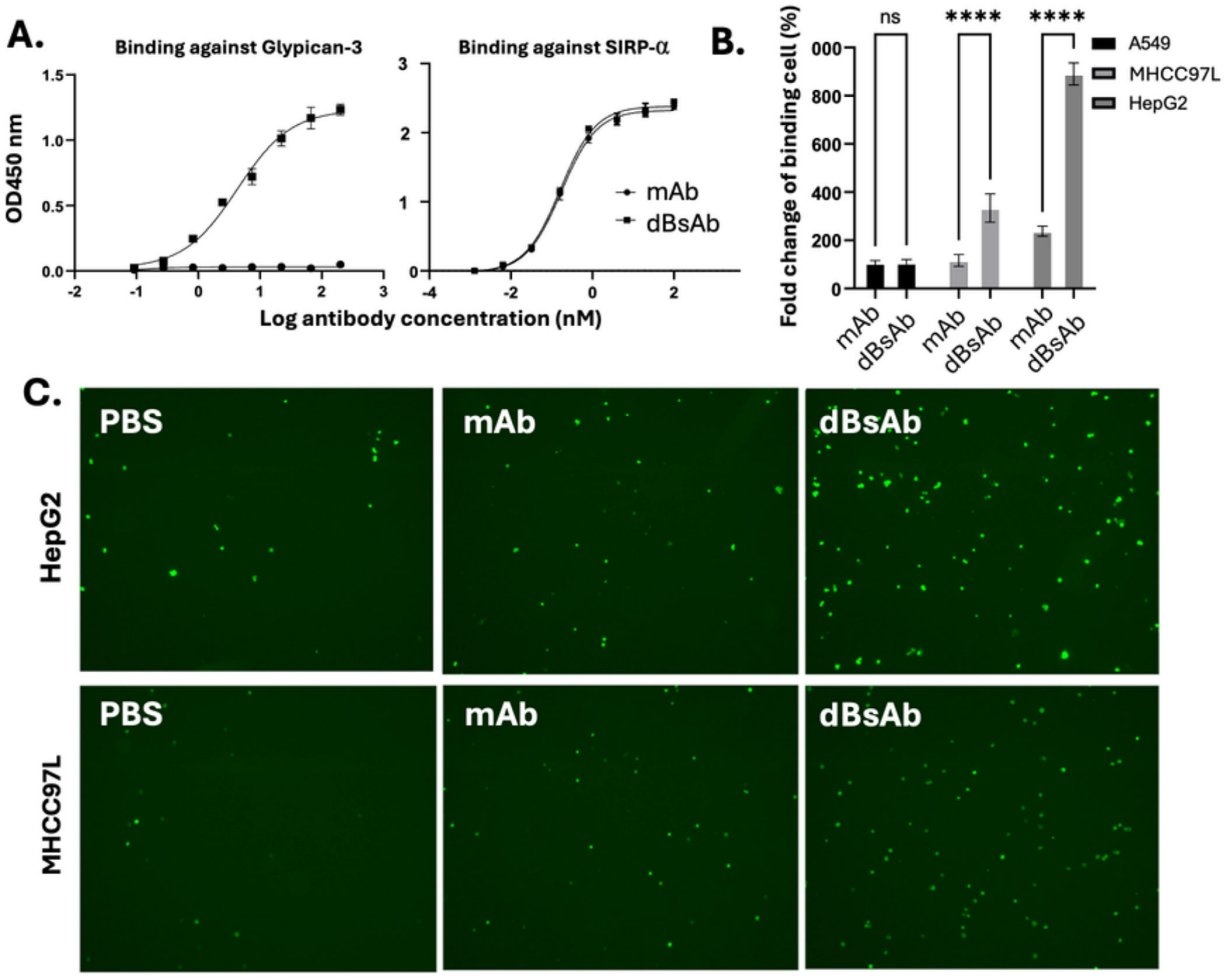
(A) ELISA (enzyme‐linked immunosorbent assay) binding assays of target binding affinity of dBsAb compared to unmodified monoclonal antibody (mAb). (B) Quantification analysis of macrophage‐liver cell adhesion, represented as percentage fold‐change relative to baseline adhesion levels in the absence of antibody. (C) Representative fluorescence microscopy images showing the green‐labeled macrophages adhered to cancer cells when treated with dBsAb or mAb. Data are expressed as the mean ± standard deviation of four independent experiments. ****p < 0.0001 as calculated by the Student's *t*‐test with *n* = 3.

Our molecular design of the novel dBsAb combines the binding ability of a monoclonal antibody and a glypican‐3 peptide (AcNH‐CALLANHEELFQT‐CONH_2_) reported by Feng et al. against the glypican‐3 receptor [44]. To join the two components, we utilized a bifunctional linker that features a 1,3‐bis(bromomethyl)benzene (BBMB) unit for thiol‐containing tumor‐targeting peptide conjugation [45, 46, 47] and an *ortho*‐phthalaldehyde (OPA) group for phthalaldehyde‐amine capture reaction [48, 49, 50, 51, 52] performed on the antibody surface. Similar methods have been used in our previous investigation and showed impressive results [52, 53, 54]. (Figure 2A). In brief, the first step involves the conjugation of glypican‐3‐targeting peptide with BBMB via thiol alkylation reaction. The reaction was performed by mixing 2 mM bifunctional linker and 4 mM glypican‐3‐binding peptide in borate buffer (50 mM, pH 10.0) for 1 h at room temperature, forming a peptide dimer. 2% TFA was then used to remove the OPA‐protecting group, followed by neutralization with 1 M NaOH (Scheme S1). The conjugation reaction was monitored using RP‐HPLC and MALDI‐TOF, achieving over 90% conversion (Figures S1 and S2). Without further purification, the resulting peptide dimer in various ratios was added to the monoclonal antibody at 0.02 mg/mL in PBS (pH 7.4) for 1 h at room temperature. Finally, the excess peptide dimer was removed using a Zeba Spin Desalting Column (40K MWCO), generating dBsAb. The modification process can be completed in less than 4 h, forming the dBsAb via a one‐pot, two‐step reaction. A monomeric bispecific antibody (mdBsAb) was also synthesized for comparison.

### 2.2 Characterization and Docking Experiment

After establishing the synthetic methodology, we determined the optimal amount of peptide dimers on one antibody. To this end, optimization was performed by reacting the antibody with 5, 10, 25, and 50 equivalents of peptide dimers, followed by characterization with SDS‐PAGE and mass spectrometry (Figure S3). Our mass spectrometry data revealed that with a 1:50 condition, it generates an antibody‐to‐peptide ratio of 1:7.3 constantly, with low batch‐to‐batch variation (Figure S4). The enzyme‐linked immunosorbent assay (ELISA) was used to determine the best reaction conditions with the highest binding affinity toward the targets (Figure S5). The data suggested that using antibodies to peptide dimers at a ratio of 1:50 for the conjugation reaction, the binding affinity against glypican‐3 was determined to be 9.51 nM, while no significant alteration was observed in the binding affinity against SIRP‐α (Figure 2A). Further increases in the peptide concentration led to precipitation and loss of binding affinity. Thus, we used a 1:50 ratio for future experiments. We further conducted a competitive ELISA to compare the glypican‐3 binding of the monomeric bispecific antibody and the dimeric dBsAb. The dBsAb exhibited significantly higher binding, consistent with avidity effects (Figure S6).

**FIGURE 3:**
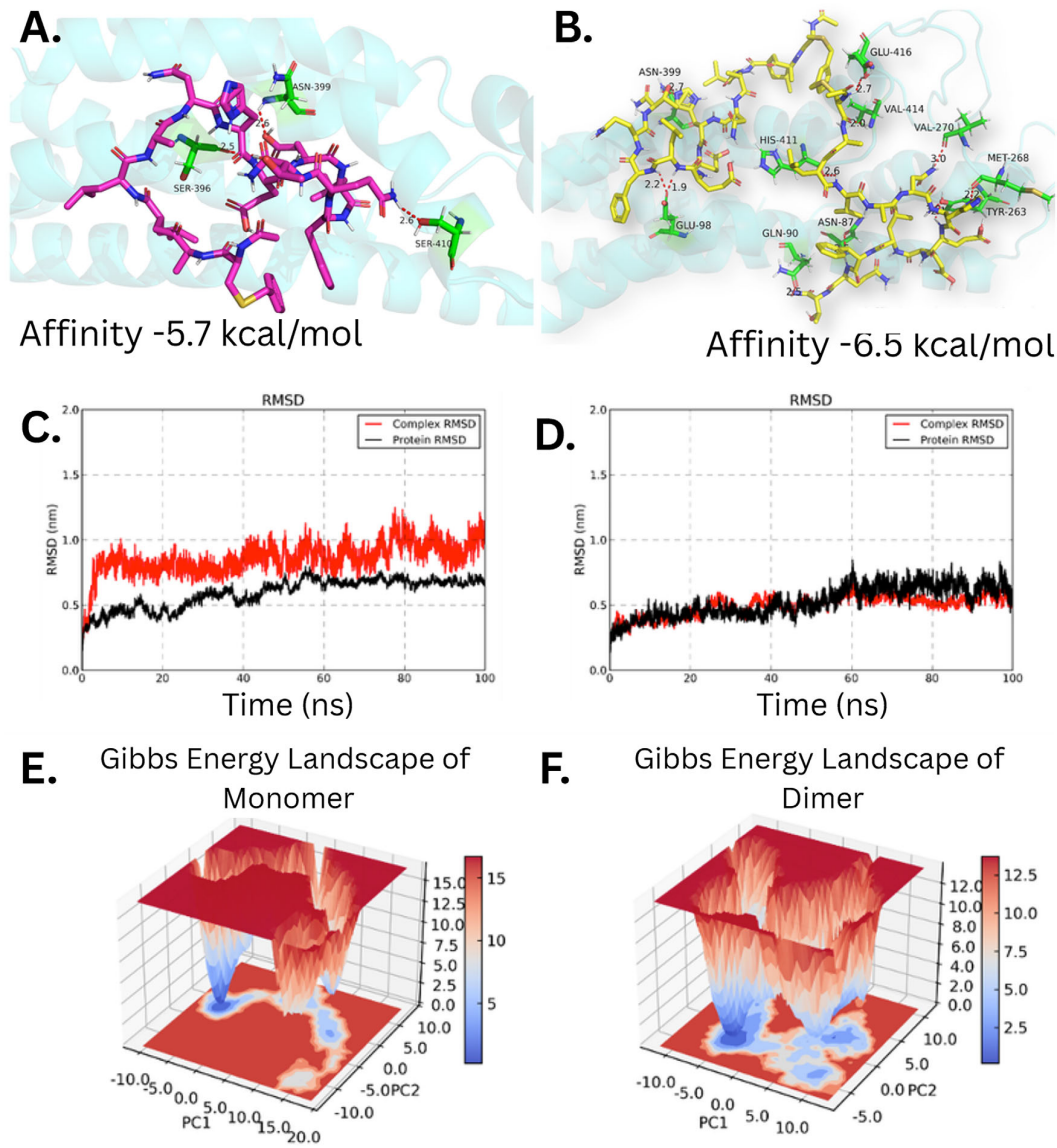
Illustration of the binding pose and energy between glypican‐3‐binding peptide (A) monomer and (B) dimer against glypican‐3 receptor. Our models show that the dimeric structure of glypican‐3‐binding peptides has a higher binding affinity against the glypican‐3 receptor compared to the monomeric counterpart. (C) and (D) show the root‐mean‐square deviation (RMSD) profile of monomer and dimer, respectively. The results illustrate that the dimer complex exhibits lower and more stable RMSD values, indicating a more stable binding mode than the monomer. (E and F) Gibbs free‐energy landscapes projected for monomer and dimer against the glypican‐3 protein, respectively. Energy values (kJ/mol) are color‐coded from low (blue, energetically favorable) to high (red). The monomer (E) shows a single deep, narrow basin, while the dimer (F) samples multiple shallow, well‐connected minima, consistent with a broader yet thermodynamically stable conformational ensemble.

A docking experiment was also conducted to confirm our design. Analysis of the binding interactions revealed that dimerization of the peptide via a thiol‐linked dimethylbenzene linker enhanced receptor engagement, as evidenced by an increased number of molecular contacts. The peptide dimer exhibited improved binding affinity of −6.5 kcal/mol and dissociation constant of 1.70 × 10^−5^ M compared to its monomeric counterpart of −5.7 kcal/mol and 6.65 × 10^−5^ M, respectively (Figure 3). We found that both monomer and dimer bind to a similar region on glypican‐3 receptor, and the 2D protein–ligand binding projection shows increased interactions between the peptide dimer and glypican‐3 receptor (Figures S7 and S8). The backbone root‐mean‐square deviation (RMSD) was calculated to evaluate the conformational stability of the complexes. As shown in Figure 3C, the monomer–protein complex exhibited pronounced fluctuations during the initial stages of the simulation, with an overall upward trend and a maximum RMSD value of approximately 10.0 Å, deviating substantially from the trajectory of the protein alone. In contrast, Figure 3D illustrates the dimer–protein complex, which displayed a smaller initial increase, reaching dynamic equilibrium during the simulation, without exhibiting significant structural distortions or abnormal energy fluctuations. The maximum RMSD value for the dimer complex was approximately 5.0 Å, indicating reduced conformational deviation compared to the monomer. Collectively, these results suggest that the dimer forms a more stable and well‐converged ligand–protein complex relative to the monomer. Free energy landscape analysis was performed using RMSD and radius of gyration (Rg) as reaction coordinates. As shown in Figure 3E, the monomer complex displayed sparse and scattered low‐energy regions, suggesting limited stabilization. In contrast, Figure 3F suggests that the dimer complex possessed enriched and concentrated low‐energy basins, reflecting its stable energy state in the complex system. These data support the use of glypican‐3‐targeting peptide dimers for antibody conjugation strategies.

### 2.3 Target Binding and Macrophage‐Engaging Properties

Following the successful optimization of the generation of dBsAb, in vitro experiments were conducted to assess the selectivity toward target cell lines. We compared glypican‐3 high‐expressing liver cancer cells, MHCC97L, with glypican‐3 low‐expressing human embryonic kidney cell line HEK293. Flow cytometry analysis demonstrated that our dBsAb exhibited high specificity toward liver cancer cells and RAW264.7 murine macrophages, confirming the bifunctionality in vitro (Figure S9). The serum stability of the dBsAb was evaluated through a serum stability assay in 50% serum, confirmed by ELISA, showing that the binding ability remained unchanged after serum treatment for up to 48 h (Figure S10). The glypican‐3 peptide dimers were determined to be non‐toxic at the highest concentration of 10 µM against all tested cell lines (Figure S11).

Subsequently, we investigated the macrophage‐engaging capability of the dBsAb using a reported method [55]. This approach examined the interaction between fully confluent cancer cell cultures and green fluorescence‐labeled macrophages incubated together for 10 min in the presence or absence of dBsAb or mAb. Through fluorescent microscopy and ImageJ analysis, the data indicated that a significantly higher number of macrophages were engaged with the liver cancer cells in the presence of dBsAb when compared to the mAb, creating new cell–cell interactions (Figure 2B,C and Figure S12). We further compared the macrophage‐engaging property of the monomeric peptide conjugate, mdBsAb, which was used. Our data showed that the dBsAb significantly enhanced macrophage adhesion to glypican‐3‐expressing liver cancer cells compared to monomeric counterparts. The increased multivalent peptide design of dBsAb promotes stronger and more efficient interaction, resulting in up to nine‐fold increase in macrophage binding, thereby supporting its superior immune recruitment potential (Figure S12). When non‐liver cancer cell lines did not show an increase in macrophage number with dBsAb or mAb. This demonstrates that dBsAb effectively links macrophages specifically to liver cancer cells in proximity while leaving the non‐liver cancer cells intact. In addition, our dBsAb also demonstrates the blockage of CD47–SIRP‐α interaction by competitive ELISA, further enhancing the phagocytic activity of macrophages (Figure S13).

### 2.4 Phagocytosis Property of dBsAb

**FIGURE 4:**
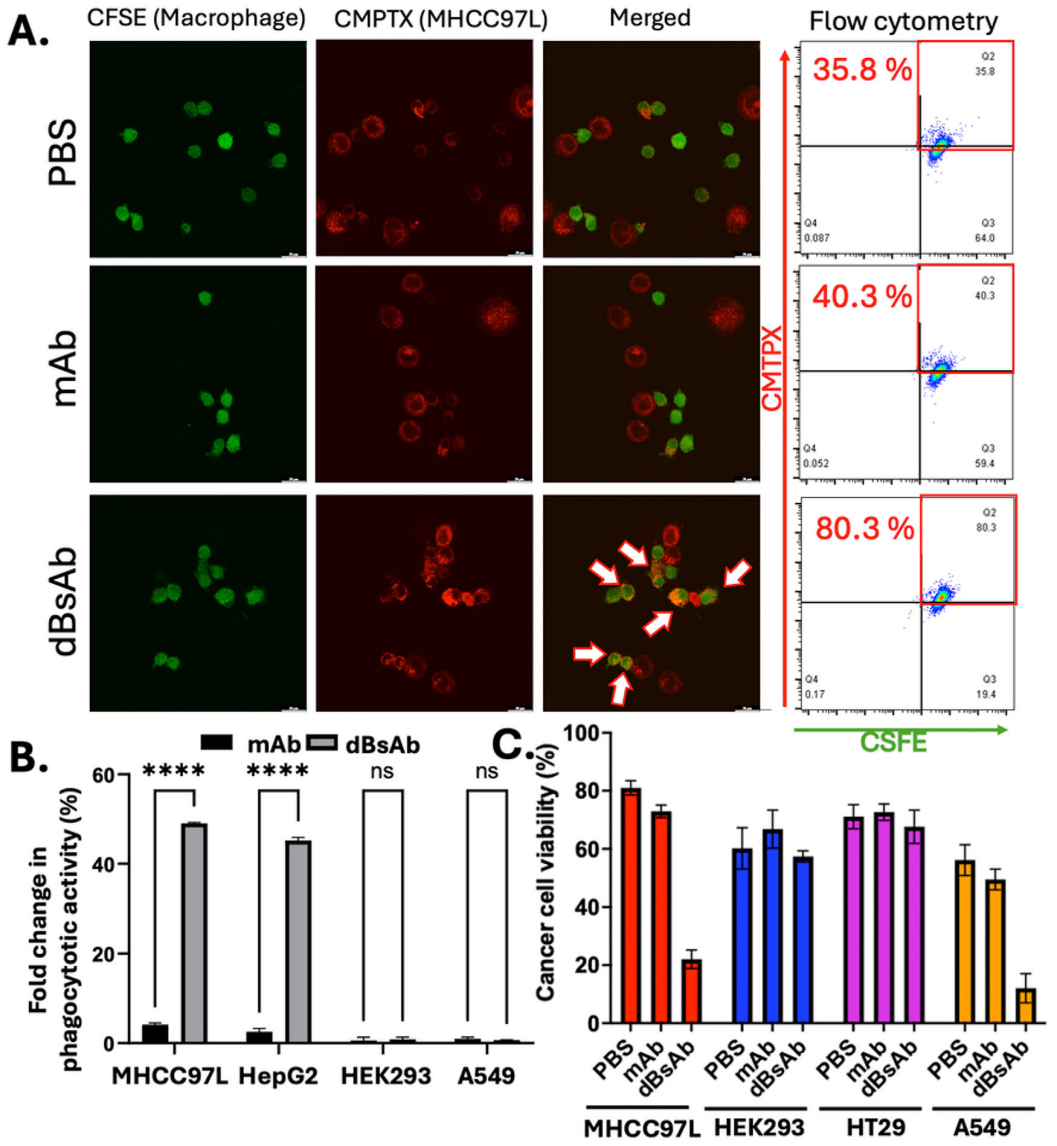
(A) Confocal microscopic images of MHCC97L cell lines (red) cocultured with RAW264.7 macrophages (green). The white arrow shows the overlapping colors (yellow) in green macrophages, indicating the phagocytic macrophage that engulfed the cancer cells. The flow cytometry data showed that the percentage of phagocytic macrophages increased 100% in the presence of dBsAb compared to mAb treatment. (B) Flow cytometry quantifies the fold‐change of phagocytic macrophages in different cell lines in the presence of mAb or dBsAb. (C) The cancer cell viability of prolonged coculture with macrophages and different cell lines in the presence of mAb or dBsAb. The data show a significant anticancer effect of macrophages in the presence of dBsAb against MHCC97L and HepG2. CSFE: CellTrace carboxyfluorescein succinimidyl ester; CMPTX: CellTracker Red. Data are expressed as the mean ± standard deviation of four independent experiments. ****p < 0.0001 as calculated by the Student's *t*‐test.

When liver cancer cells and macrophages are allowed to interact for an extended period, phagocytosis occurs. To determine the effect of dBsAb on enhancing macrophage phagocytosis, we utilized flow cytometry and confocal microscopy to capture the phagocytosis process of macrophages. For confocal microscopic studies, red fluorescence‐labeled cancer cells and green fluorescence‐labeled RAW264.7 macrophages were cocultured in confocal dishes for 12 h in the presence of mAb or dBsAb. The white arrows in Figure 4A specify the macrophages that contain both green fluorescence from the macrophages and red fluorescence from the cancer cells, indicating their phagocytic activities against cancer cells. Our data demonstrated that in the presence of dBsAb, the number of double‐positive macrophages was significantly higher than that in treatment with mAb and PBS (Figure 4A and Figure S14), suggesting that dBsAb enhances the liver cancer‐specific phagocytosis activity of macrophages.

Quadrant analysis results of the macrophage populations of flow cytometry also illustrated that the double‐positive region increased in the presence of dBsAb, reflecting the number of macrophages that have phagocytosed liver cancer cells (Figure S15). For glypican‐3‐overexpressing cells MHCC97L, the percentage of phagocytic macrophages slightly increased from 35.8% with PBS to 40.3% with mAb and significantly increased to 80.3% with dBsAb (Figure 4B). A similar trend was observed in HepG2 cells, with the phagocytic macrophage amount increasing from 21.8% with PBS to 25.0% with mAb, and 67.1% with dBsAb. However, non‐liver cancer cell lines such as HEK293 and A549 showed no significant enhancement in phagocytosis, even in the presence of dBsAb compared to controls (Figure 4B). Whereas minimal phagocytosis was detected in the PBS and monomeric peptide conjugate groups (Figure S16). We suspected such dramatic improvement between monomer and dimer conjugates was mainly driven by doubling the number of peptides on an antibody surface. Thus, the enhanced avidity boosts local ligand concentration, promotes rebinding to glypican‐3, and reduces dissociation. Docking studies also revealed that extra interactions with the receptor may play a role in the enhanced phagocytic effect. Collectively, the higher avidity and additional receptor contacts likely account for the increased phagocytic activity observed with the dimer conjugate.

Cancer cell viability was also performed by Cell‐Titer Glo assay, and the data revealed the enhanced anticancer effect of macrophages in the presence of dBsAb (Figure 4C). After that, we would like to investigate if the treatment could also enhance the antigen presentation process of macrophages, which is an important factor in immunotherapy. Thus, we performed quantitative polymerase chain reaction (qPCR) to assess the antigen presentation activity of macrophages after phagocytosis by measuring the expression of the *H2‐Eb1* gene, which is responsible for the upregulation of MHC Class II receptors involved in antigen presentation. Our results showed that upregulation of the *H2‐Eb1* gene in macrophages occurred only when coculturing with glypican‐3‐overexpressing HepG2 cells in the presence of dBsAb (Figure S17). This result indicates that the dBsAb promotes both phagocytosis and antigen presentation, the latter being important for engaging adaptive immune responses against tumors.

### 2.5 In Vivo Anticancer Property, Toxicity, and Pharmacokinetic Studies of dBsAb

**FIGURE 5:**
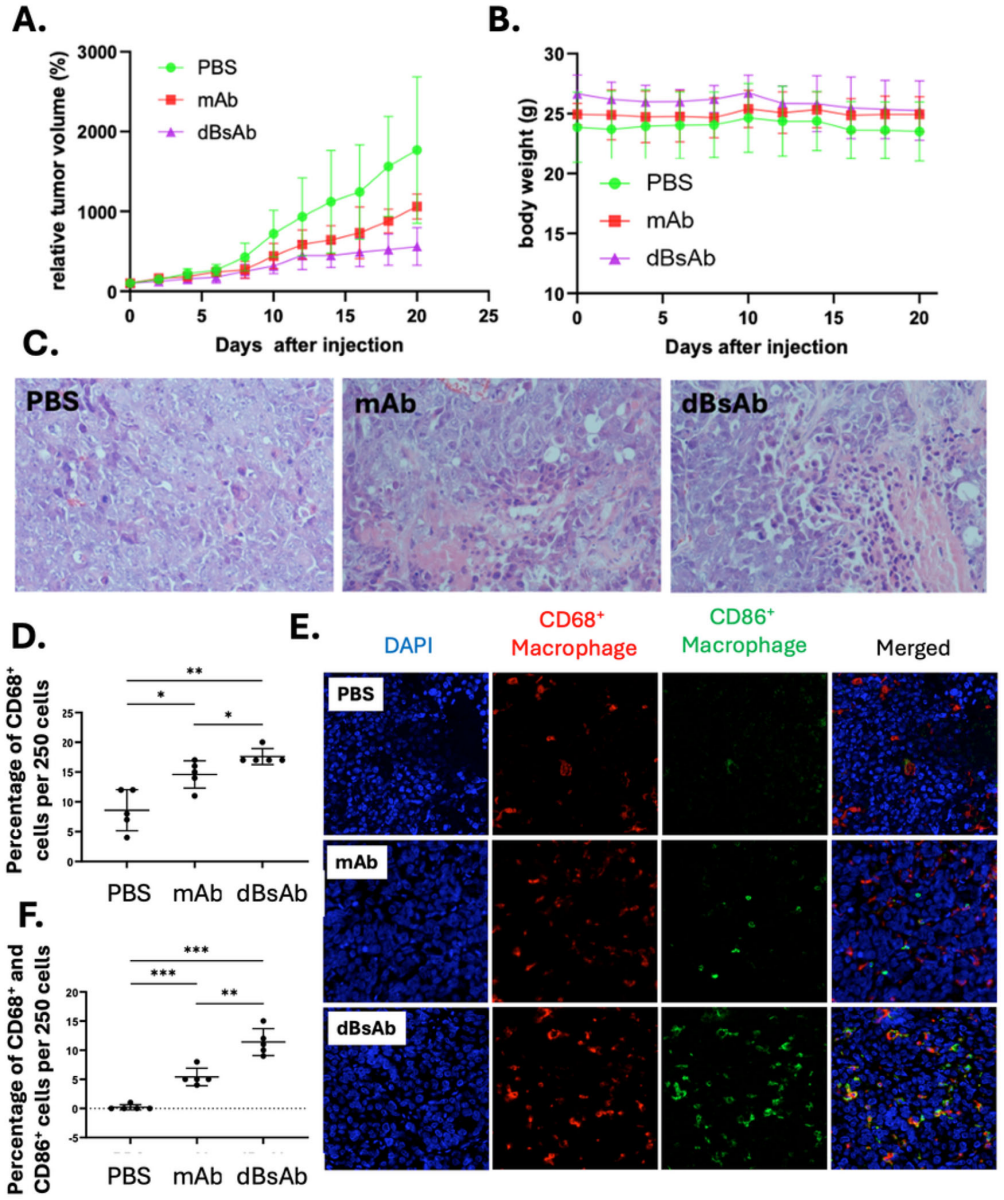
(A) Tumor growth curves over 20 days (*n* = 6). (B) Bodyweight changes under different treatments (*n* = 6). (C) Hematoxylin and eosin (H&E) and macrophage staining of dissected tumor tissues. (D) Quantification of CD68^+^ macrophage infiltration. (E) Multiplex immunofluorescent staining of CD86^+^ and CD68^+^ macrophages with *n* = 5. (F) Quantification of CD68^+^/CD86^+^ M1 macrophages. Data are expressed as the mean ± standard deviation of four independent experiments. *p < 0.05; **p < 0.01; ***p < 0.001 as calculated by the Student's *t*‐test.

To validate the efficacy of dBsAb in vivo, we conducted in vivo experiments to determine the therapeutic efficacy of our first‐in‐class macrophage engager. MHCC97L xenografted nude mice were administered three doses of 3 mg/kg in 100 µL of dBsAb, mAb, or PBS at Days 0, 7, and 12 via intravenous injection. Tumor size and bodyweight were recorded over 20 days. On Day 20, tumors were harvested for hematoxylin and eosin (H&E) histology analysis and multiplex immunofluorescence staining to assess the change in macrophage number and their activation status inside the tumor under the treatment of dBsAb or mAb. Control groups underwent identical staining procedures for comparison. Our results demonstrated that treatment with dBsAb significantly reduced 68.33% of the tumor sizes in the nude mice model, while only 40.07% inhibition was observed for monoclonal antibody treatment compared to PBS (Figure 5A). The stable trends of bodyweight showed the low toxicity of dBsAb (Figure 5B). The H&E staining images show the histological evaluation of tumor tissues treated with PBS, mAb, and dBsAb. The PBS‐treated section shows dense tumor cells with minimal immune cell infiltration, indicating active tumor growth. In the mAb‐treated section, some immune cell infiltration and partial tumor cell disruption can be observed, showing moderate efficacy. In contrast, the dBsAb‐treated section exhibits substantial immune cell infiltration and extensive tumor cell damage, suggesting enhanced tumor clearance and effective macrophage‐mediated antitumor activity by the dBsAb treatment (Figure 5C).

To further investigate the anticancer mechanism of dBsAb, multiplex immunofluorescent staining was performed to determine the macrophage infiltration and activation. To our surprise, we found that the number of CD68^+^ macrophages inside the tumor sections increased significantly compared to the PBS control. This revealed that the dBsAb could promote macrophage infiltration into solid tumors (Figure 5D and Figure S18). Interestingly, when co‐staining CD68 with CD86, which is an M1 marker of macrophage, significant enhancement of the numbers of M1 macrophages was observed inside tumor sections only under the treatment of dBsAb (Figure 5E,F). Therefore, we concluded that in the presence of the SIRP‐α mAb or dBsAb, both could cause enhanced macrophage infiltration into solid tumors, and thus, the regression of tumor volume was observed. However, dBsAb not only blocked the “don't eat me” signal but also could even activate the macrophages, turning them into an M1 state. Thus, dBsAb could further reduce the tumor volume significantly compared to the mAb treatment.

**FIGURE 6:**
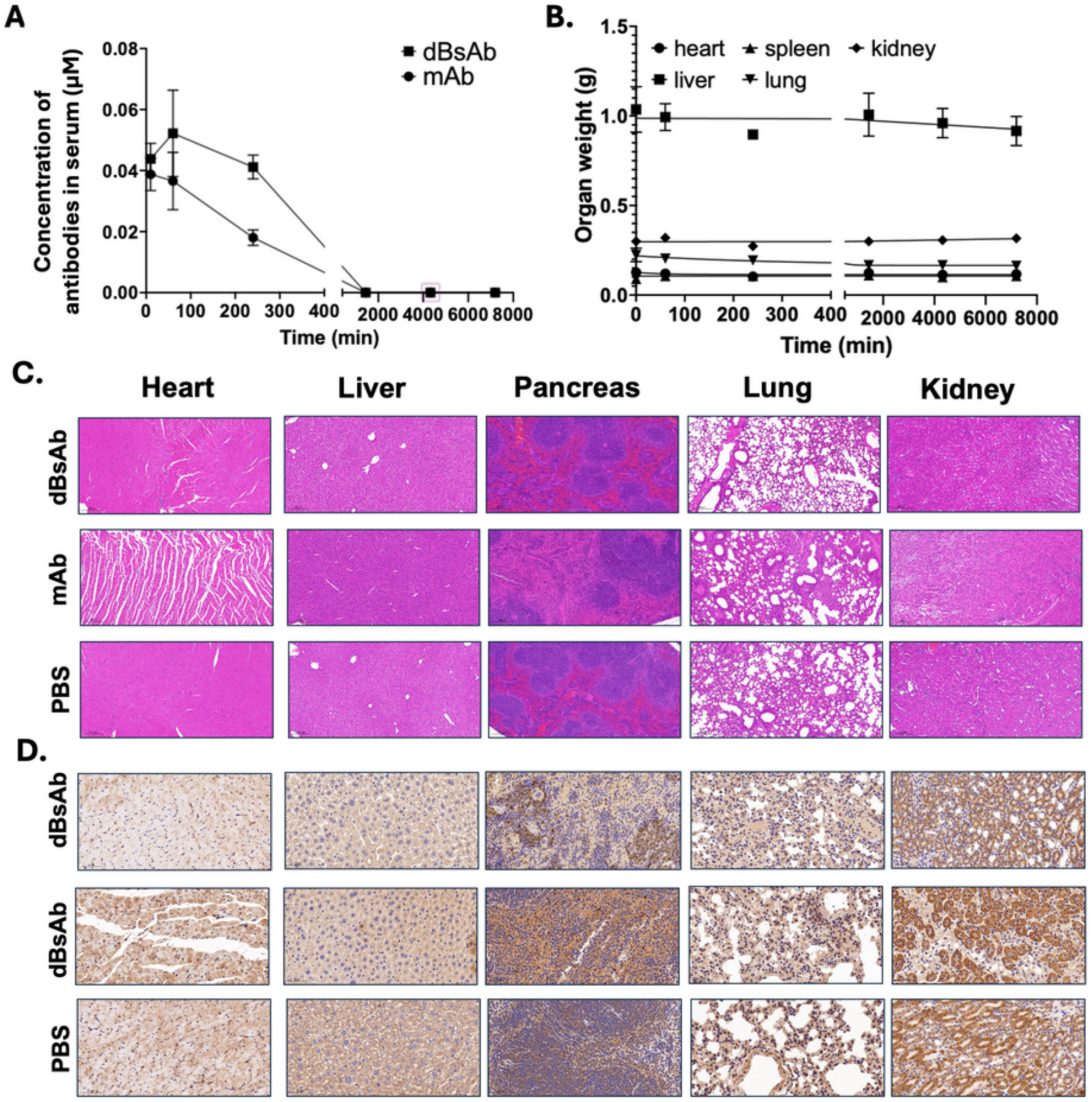
The pharmacokinetic and toxicology studies of the dBsAb compared to unmodified mAb. (A) Serum concentrations of each antibody are measured at the indicated time points using an anti‐rabbit IgG sandwich ELISA (enzyme‐linked immunosorbent assay). The plotted data represent mean ± SEM. (B) Organ weights of major tissues—including heart, liver, spleen, lung, and kidney—are recorded at various time points (0 to 7200 min) following intravenous injection of dBsAb (5 mg/kg, *n* = 3 per time point). No significant changes in organ weight are observed throughout the study period, indicating an absence of gross organ toxicity. Data represent mean ± SEM. These findings support the tolerability of dBsAb construct in vivo. (C) Hematoxylin and eosin staining of different organs after treatment with different antibodies against PBS control after 5 days post 10 mg/kg intravenous injection. (D) Tumor necrosis factor alpha (TNF‑α) immunohistochemical staining in heart, liver, spleen, lung, and kidney from mice receiving systemic administration of the monoclonal antibody or dBsAb, collected at 5 days post dose, compared with untreated controls.

To assess the impact of the structural modifications on serum stability, we compared the pharmacokinetic profiles of the modified bispecific antibody (dBsAb) against the native monoclonal antibody (mAb) following intravenous administration. Female BALB/c mice (9–11 weeks old) were administered a single intravenous dose of 5 mg/kg dBsAb via the tail vein. At predetermined time points (10, 60, 240, 1440, 4320, and 7200 min post‐injection), groups of mice (*n* = 3 per time point) were euthanized with ketamine and xylazine, followed by cardiac puncture for whole blood collection. Serum was isolated by centrifugation at 4000 × *g* for 15 min at 4°C, followed by rapid flash‐freezing in liquid nitrogen, and stored at –80°C until analysis. The samples were diluted 5000‐fold, and the concentration of dBsAb in mouse serum was quantified by a sandwich ELISA. Immediately following administration, the dBsAb achieved a higher maximum serum concentration of approximately 0.055 nM, whereas the native mAb showed a lower initial recovery. This differential suggests that the dBsAb may possess enhanced resistance to immediate proteolytic degradation or rapid tissue distribution upon entry into the systemic circulation. During the initial elimination phase (0–60 min), the native mAb underwent rapid clearance, with serum levels dropping precipitously to approximately 0.02 nM. In contrast, the dBsAb demonstrated enhanced retention, maintaining a concentration of roughly 0.035 nM at the 60‐min mark. This represents a retention of approximately 63% of the initial dose, significantly higher than that of the native control. Throughout the intermediate phase (60–240 min), the dBsAb consistently maintained higher serum levels than the native mAb. Although both constructs displayed first‐order elimination kinetics, the rate of decline for the dBsAb was notably slower. By 240 min, the dBsAb concentration remained detectable at approximately 0.01 nM, while the native mAb approached the lower limit of quantification. Both profiles converged near zero by the 400‐min mark, indicating complete clearance (Figure 6A). No significant changes in major organ weights were observed throughout the study, indicating no apparent systemic toxicity (Figure 6B). H&E staining and tumor necrosis factor‐alpha (TNF‐α) immunohistochemistry (IHC) were performed on major organs after 5 days of treatment with 10 mg/kg dBsAb, revealing no histopathological abnormalities or inflammatory damage (Figure 6C,D).

## 3 Discussion

Although bispecific antibodies have shown considerable promise in cancer therapy, their translation adaptation has been hindered by challenges such as difficult R&D initiation, complex recombinant production, and scalability. Conventional approaches that rely on extensive cell line engineering to assemble heterodimeric proteins result in high cost, long development cycles, and manufacturing inefficiencies. In this work, we used a chemical conjugation strategy that can generate a bispecific antibody from any readily available monoclonal antibodies. Central to this approach is the rational design of a bifunctional linker that on one side contains a BBMB moiety for rapid and selective reactivity toward thiol groups, allowing efficient covalent coupling with cysteine‐containing peptides under mild aqueous conditions. This reaction forms peptide dimers on one linker, which enhances target binding through increased avidity. The second functional moiety is the OPA that enables conjugation to surface‐exposed amine residues on the antibody. We designed to use the OPA‐amine capture reaction rather than other commonly used amine‐reactive groups, such as *N*‐hydroxysuccinimide (NHS) esters, because usually they are susceptible to hydrolysis in aqueous buffer and often result in reduced coupling efficiency. On the other hand, OPA remains stable in aqueous buffer and in the absence of amines, and they do not generate side‐products after reaction. This stability reduces the need for intermediate purification and simplifies the overall assembly workflow.

In this proof‐of‐concept research, we focus on macrophage engagement rather than the extensively studied T‐cell redirection approach in most bispecific antibodies on the market. We want to leverage the unique immunological importance of these professional antigen‐presenting macrophages. While T‐cell engagers require pre‐existing tumor‐reactive T cells and function primarily through direct cytotoxicity, macrophages serve as orchestrators of both innate and adaptive immunity. Following phagocytosis, macrophages process tumor antigens and present them via MHC Class II molecules to CD4^+^ helper T cells and cross‐present via MHC Class I to CD8^+^ cytotoxic T cells, acting as an in situ source of tumor antigen presentation. The enhanced 4.8‐fold H2‐Eb1 expression was observed, confirming the upregulation of antigen presentation machinery, amplifying antitumor immunity, and potentially generating immunological memory against multiple tumor neoantigens simultaneously. The design of the first target of our first dBsAb aims to disrupt the CD47–SIRP‐α checkpoint at the same time, being an anchor point on the immune cell, while the second target ensures tumor selectivity through multivalent glypican‐3 recognition. This dual‐action mechanism translated into measurable functional gains, with phagocytic efficiency reaching 80.3% compared to 40.3% observed with unmodified SIRP‐α antibody, translating directly to 68% tumor volume reduction at a modest 3 mg/kg dosing regimen—lower than many reported antibody dosing regimens.

Further insight into the immunological effects of dBsAb came from tumor microenvironment analysis. Multiplex immunofluorescence revealed not merely increased CD68^+^ macrophage infiltration but also a shift toward the M1 phenotype, as indicated by increased CD86 expression. M1‐polarized macrophages produce proinflammatory cytokines and can recruit additional immune effectors to the tumor site. Unlike T‐cell engagers that create binary kill‐or‐escape outcomes, macrophage‐mediated tumor destruction generates an immunogenic cell death that releases damage‐associated molecular patterns (DAMPs), further amplifying the immune response. Such a cascade of innate‐to‐adaptive immune activation could help overcome the immunosuppressive microenvironment of poorly immunogenic tumors.

Despite using immunocompromised nude mice that lack functional T cells and adaptive immunity, our dBsAb achieved a remarkable 68% tumor reduction, demonstrating the potent direct effects of macrophage‐mediated phagocytosis alone. Notably, nude mice retain only innate immune components—primarily macrophages, neutrophils, and NK cells—indicating that the observed efficacy reflects innate immunity alone. In immunocompetent models with intact T‐cell populations and fully functional antigen presentation pathways, the addition of adaptive immune responses in immunocompetent hosts may further improve efficacy, including cytotoxic T‐cell activation, helper T‐cell support, and B‐cell‐mediated antibody production. These results support further evaluation in syngeneic or humanized mouse models where the full spectrum of macrophage‐initiated immunity can be evaluated. Meanwhile, our chemical assembly platform enables rapid exploration of alternative target combinations beyond glypican‐3 and SIRP‐α, with multiple candidates currently under investigation. As these studies progress through our screening pipeline, we will report findings that further validate this approach for addressing diverse cancer types and overcoming specific resistance mechanisms in due course.

The pharmacology studies determine if the conjugation of glypican‐3 peptide dimers to the antibody scaffold would compromise systemic stability compared to the native monoclonal antibody. Despite the increased structural complexity, the dBsAb showed improved serum retention compared with the unmodified mAb. The extended half‐life and higher area under the curve observed for the dBsAb imply that the structural modifications may offer a protective effect. This could be attributed to steric hindrance preventing access by serum proteases or a reduction in nonspecific binding to endothelial surfaces. The rapid clearance of the native mAb is consistent with typical clearance mechanisms for unmodified IgG in murine models, consistent with the improved retention profile of the conjugated construct.

In addition to the promising results demonstrated in this study, there are methodological limitations inherent to our current chemical conjugation approach that warrant discussion. The bispecific antibody was generated through random lysine‐based conjugation, a strategy that, while efficient and mild, inevitably introduces molecular heterogeneity due to the stochastic modification of multiple surface‐exposed lysine residues. This heterogeneity results in variable peptide‐to‐antibody ratios and undefined conjugation sites. Nevertheless, it is important to note that many clinically approved antibody–drug conjugates (ADCs), such as the top‐selling ADC, trastuzumab‐emtansine (Kadcyla), also employ lysine‐based conjugation strategies and have demonstrated robust clinical efficacy and manufacturability despite their inherent heterogeneity [56]. We intentionally selected this lysine‐directed chemistry because it enables rapid, one‐step conjugation under non‐reducing conditions, preserving the antibody's structural integrity and Fc functionality while achieving higher peptide loading compared with cysteine‐based methods. Although site‐specific conjugation techniques such as glycan remodeling or incorporation of unnatural amino acids can yield more homogeneous products [57, 58], these approaches remain technically demanding and cost‐prohibitive for scalable production. Furthermore, another limitation of the current work lies in the restricted availability of tumor‐selective peptide binders, which limits the diversity of potential bispecific designs. Future integration of AI‐assisted peptide discovery and computational binder prediction could accelerate the identification of novel high‐affinity tumor‐targeting sequences, expanding the versatility and translational potential of this chemical bispecific assembly platform.

## 4 Conclusion

In summary, the chemically assembled glypican‐3 × SIRP‐α dendritic bispecific antibody described herein demonstrates a chemical approach to constructing macrophage‐redirecting bispecific antibodies. By uniting tumor‐selective glypican‐3 ligands with an anti‐SIRP‐α IgG through a one‐pot click‐chemistry protocol, we create a highly multivalent construct that overcomes the “don't eat me” barrier, drives robust phagocytosis, and elicits durable antitumor immunity in hepatocellular carcinoma models with unnoticeable organ damage. The novel protein engineering strategy delivers gram‐scale material within hours and can be re‐tooled to virtually any antigen/checkpoint pair, without the use of genetic re‐engineering. The simplicity and modularity of this platform may support its adaptation to other antigen–checkpoint combinations beyond liver cancer.

## 5 Materials and Methods

### 5.1 Preparation of Glypican‐3 × SIRP‐α dBsAb and mdBsAb

The bifunctional linker (2.0 mM) was dissolved in borate buffer (50 mM, pH 10.0) and reacted with the glypican‐3‐binding peptide (4.0 mM) for 1 h at room temperature to form the peptide dimer (A) or monomer (B). The reaction mixture was then treated with 2% TFA to remove the *ortho*‐phthalaldehyde (OPA) protecting group, followed by neutralization with 1 M NaOH to pH 7.4. The rabbit anti‐SIRP‐α monoclonal antibody (Sino Biological, China, Cat. No. 50956‐R001) (0.2 mg/mL) was diluted in PBS (pH 7.4) to a final concentration of 0.02 mg/mL. Without further purification, the peptide dimer solution was added to the antibody solution at various molar ratios of antibody to dimer = 1:5, 1:10, 1:25, 1:50, and 1:100) and reacted for 1 h at room temperature. The conjugation processes were monitored by ESI‐MS analysis. The reaction mixture was filtered through a Zeba Spin Desalting Column (40K MWCO) by centrifugation at 1500 × *g* for 2 min to remove excess unconjugated peptide dimer, yielding the purified glypican‐3 × SIRP‐α dBsAb. The final product was lyophilized and stored as a powder at −20°C until further use.

### 5.2 ELISA for Binding Analysis of dBsAb to Glypican‐3 and SIRP‐α and CD47–SIRP‐α Blockade

NUNC Maxisorp plates (Thermo Fisher Scientific) were coated with equimolar amounts of glypican‐3 (Sino Biological, China, Cat. No. 10088‐H08H) or SIRP‐α protein (Sino Biological, China, Cat. No. 50956‐M08H) and incubated overnight at 4°C. The plates were washed three times with PBS containing 0.05% Tween‐20 and blocked with 2% bovine serum albumin (BSA) in PBS with 0.05% Tween‐20 at room temperature for 1 h. For the competitive ELISA demonstrating glypican‐3 protein binding of mdBsAb and dBsAb with or without monomeric peptide blocking, 0.1 mM monomeric peptide (AcNH‐ALLANHEELFQT‐CONH_2_) in 2% BSA in PBS with 0.05% Tween‐20 was used for the blocking. For ELISA against glypican‐3, a three‐fold serial dilution of dBsAb or mAb, starting at 200 nM, was added to the plates and incubated for 2 h at room temperature. For ELISA against SIRP‐α protein, a five‐fold serial dilution of dBsAb or mAb, starting at 100 nM, was added to the plates and incubated for 2 h at room temperature. For the CD47–SIRP‐α blockade study, a five‐fold serial dilution of dBsAb, starting at 20 nM, was mixed with a fixed amount of His‐tagged CD47 protein (40 nM) (Sino Biological, China, Cat. No. 57231‐M08H) and added to the plates, followed by incubation for 2 h at room temperature. After incubation, the plates were washed three times with PBS containing 0.05% Tween‐20 and treated with horseradish peroxidase (HRP)‐conjugated goat anti‐rabbit secondary antibody (Biolegend, AB_2099368) or HRP‐conjugated anti‐His‐tag secondary antibody (Sino Biological, China, AB_2857925) diluted 1:2000 in blocking buffer for 1 h at room temperature. Subsequently, 100 µL of TMB 3,3ʹʹ,5,5ʹʹ‐tetramethylbenzidine (TMB) two‐component substrate (Biogene, China) was added to each well and incubated for 6 min, after which the reaction was stopped with HCl. Absorbance was measured at 450 nm using a plate reader. The half‐maximal effective concentration (EC_50_) for binding affinity was calculated using non‐linear regression analysis of the binding curves in GraphPad Prism.

### 5.3 Flow Cytometric Analysis of Cellular Binding of Antibodies

Approximately 2 × 10^5^ HEK293, RAW264.7, or MHCC97L cells were placed in centrifuge tubes and rinsed with 1% BSA in PBS. Then, the cells were incubated with 50 nM dBsAb or the native mAb in a medium at 4°C for 1 h, followed by rinsing with 1% BSA in PBS twice. Alexa 488‐conjugated anti‐rabbit secondary antibody (ITK Southern Biotech, China, AB_2795961) was incubated with the cells at a 1:500 dilution for 30 min. The cells were washed with 1% BSA in PBS twice and then resuspended in PBS (1.0 mL). The intracellular fluorescence intensities were measured using a BD Accuri C6 Flow Cytometer (Becton Dickinson) with 1 × 10^4^ cells counted in each sample. The dye was excited by an argon laser at 488 nm, and the emitted fluorescence was monitored at 500–560 nm. The data collected were analyzed using FlowJo. All experiments were performed in triplicate.

### 5.4 Cell–Cell Adhesion Assay

Approximately 2 × 10^5^ A549, MHCC97L, and HepG2 cancer cells per well were seeded in a 12‐well cell culture plate and incubated at 37°C in a humidified 5% CO_2_ atmosphere for 3 days, until reaching over 90% confluence. RAW264.7 macrophages (2 × 10^5^ cells per well) were first labeled with carboxyfluorescein succinimidyl ester (CFSE), followed by washing with PBS and premixing with 50 nM of dBsAb, mAb, or mdBsAb, before adding to cancer cells. The cells were allowed to interact for 10 min in complete medium at 37°C in a humidified 5% CO_2_ atmosphere. After incubation, the wells were thoroughly washed three times with PBS. The cells were subsequently examined using a ZEISS Avio Vert.A1 fluorescence microscope, and the images were analyzed with ImageJ software to quantify the number of CFSE‐labeled macrophages remaining on the confocal dish. The cells were counted manually by using the Cell Counter function of ImageJ. The experiment was performed in quintuplicate. The fold‐changes of binding cells relative to baseline adhesion levels in the absence of antibody were calculated according to the equation below:

$$\frac{\mathrm{Number}\;\mathrm{of}\;\mathrm{binding}\;\mathrm{cells}\;\mathrm{with}\;\mathrm{dbsAb}\;\mathrm{or}\;\mathrm{mAb}\;\mathrm{treatment}}{\mathrm{Number}\;\mathrm{of}\;\mathrm{binding}\;\mathrm{cells}\;\mathrm{with}\;\mathrm{PBS}\;\mathrm{treatment}}\times 100$$

### 5.5 Molecular Docking Experiment

The 13‐mer linear glypican‐3 binding peptide (AcNH‐CALLANHEELFQT‐COHN_2_) was selected for computational assessment of modified glypican‐3 binding peptide affinity with glypican‐3 protein. This peptide was chosen because it was previously reported for its glypican‐3 specificity using phage display. Two structures of modified peptide, which are bromomethyl benzene‐glypican‐3 binding peptide and dibromomethyl benzene‐glypican‐3 binding peptide, were drawn on ChemDraw version 22.0. The crystallographic coordinate of the glypican‐3 was obtained from the protein data bank with the PDB ID 7ZA2 (resolution: 4.60 Å). The protein structure was prepared on PyMOL (the Molecular Graphics System, Version 3.0, Schrödinger LLC.). Chain B, C, D, E, F, G, H from the complex 7ZAV2 were removed, as well as water and NAG. Energy minimization was conducted on ChemDraw 3D version 22.0 to achieve the lowest energy from the docking. The ligand–protein docking was performed using AutoDock Vina version 1.5.7. The grid box was set by covering the whole protein area. The conformations and poses with the highest binding affinity were selected and exported to PyMOL. The protein–ligand complexes were generated, and the interactions were visualized using PyMOL and LigPlot+ version 2.2.9. The distance cutoffs for polar and hydrophobic interactions were established at 3.5 and 4.0 Å, respectively.

### 5.6 Confocal Microscopic Analysis of Antibody‐Dependent Cellular Phagocytosis

RAW264.7 macrophages were harvested by washing the adherent cells with 10 mL of cold PBS, incubating for 10 min, and gently scraping to detach them. Macrophages (2 × 10^5^ cells per dish) were labeled with CFSE, while A549, MHCC97L, and HepG2 cancer cells (1 × 10^5^ cells per dish) were stained with CellTracker Red CMPTX following the manufacturer's protocol. Both cell types were then combined and seeded in a confocal dish in the presence of either 50 nM dBsAb or mAb and incubated for 12 h at 37°C in a complete medium. After incubation, the medium was removed, and the cells were washed twice with PBS before being examined using a Leica TCS SP8 high‐speed confocal microscope equipped with solid‐state lasers. Fluorescence was monitored at excitation wavelengths of 488 and 577 nm. The images were digitized and analyzed using Leica Application Suite X software.

### 5.7 Flow Cytometric Analysis of Antibody‐Dependent Cellular Phagocytosis

RAW264.7 macrophages were labeled with CFSE, while A549, MHCC97L, HepG2, and HEK293 cells were stained with CellTracker Red CMPTX according to the manufacturer's instructions. The macrophages (1 × 10^4^ cells per well) and cancer cells (1 × 10^4^ cells per well) were then incubated together in a 96‐well U‐bottom plate in the presence of dBsAb or mAb (50 nM) for 2 h at 37°C in a complete medium. After incubation, the cells were fixed with 4% paraformaldehyde for 20 min and then washed three times with PBS. The intracellular fluorescence was measured using a BD Accuri C6 flow cytometer (Becton Dickinson) with 1 × 10^4^ cells counted in each sample. The data collected were analyzed using FlowJo. All experiments were performed in triplicate. The phagocytic activity was calculated according to the equation below:

$$\frac{\mathrm{Phagocytic}\;\mathrm{macrophages}\;\left(\mathrm{double}\;\mathrm{positive}\right)}{\mathrm{Total}\;\mathrm{macrophages}}\times 100\%$$

### 5.8 CellTiter‐Glo Luminescent Cell Viability Assay

Approximately 1 × 10^4^ HepG2, HT29, HEK293, or MHCC97L cells and 2 × 10^4^ RAW264.7 macrophages per well were mixed in white 96‐well plates in the presence of 50 nM dBsAb or mAb, and the cells were cocultured overnight. At the same time, the wells with only cancer cells were prepared as controls. After overnight incubation, 50 µL of CellTiter Glo (Promega, USA) was added to each well. The luminescence signals were measured using a Thermo Fisher Scientific Varioskan LUX Multimode Microplate Reader. All experiments were performed in triplicate. The cell viability was calculated according to the equation below:

$$\left(1-\frac{\mathrm{Untreated}\;\mathrm{coculture}\;\mathrm{sample}-\mathrm{dBsAb}\;\mathrm{or}\;\mathrm{mAb}\;\mathrm{treated}\;\mathrm{coculture}\;\mathrm{sample}}{\mathrm{Untreated}\;\mathrm{cancer}\;\mathrm{cell}\;\mathrm{only}\;\mathrm{sample}}\right)\times 100\%$$

### 5.9 In Vivo Antitumor Efficacy

All animal experiments were approved by the Animal Ethics Committee of the Hong Kong Polytechnic University (20‐21/154‐ABCT‐R‐GRF). Female BALB/c nude mice (4–5 weeks old) were purchased from Hong Kong Polytechnic University. MHCC97L cells (1 × 10^5^ in 100 µL DMEM with 50% Matrigel base membrane [Corning Biocoat]) were subcutaneously injected at the back of the mice. When the tumor volume reached approximately 80–100 mm^3^, the mice were randomly divided into three groups (*n* = 4 per group): PBS, mAb (3 mg/kg), and dBsAb (3 mg/kg) in a double‐blind manner. The treatments were administered to the tumor‐bearing mice via intravenous injection on Days 0, 7, and 12. Tumor volume and bodyweight were measured every 2 days by blinded investigators. Tumor length and width were measured using a micrometer digital caliper (SCITOP Systems), and the tumor volume (mm^3^) was calculated using the formula: Tumor volume = [Length × Width^2^]/2. At the end of the study, the mice were euthanized, and tumors were excised, weighed, and photographed.

### 5.10 Immunohistochemistry and Immunofluorescence

Tumors were fixed in 4% paraformaldehyde for 24 h and transferred to 70% ethanol. Then the tissues were dehydrated by 95% ethanol twice for 0.5 h, and xylene twice for 1 h, followed by soaking in paraffin overnight. Then the tumors were embedded in paraffin and cut into 4‐µm sections. Before staining, sections were dewaxed in xylene and rehydrated in decreasing graded alcohols and distilled water. For H&E staining, tumor sections were stained with hematoxylin solution (Phygene, PH0515) for 1 min and were then rinsed in tap water until the water was colorless. Next, the sections were differentiated by a differentiation solution (Servicebio, G1039) for 30 s and blued by a bluing buffer for 1 min. Finally, the sections were stained with eosin stain (Phygene, PH0516) for 1 min and then rinsed in tap water until the water was colorless. Mounted sections were imaged by an Olympus CKX53 Inverted Microscope. For multiplexed fluorescent immunohistochemistry, the tyramide signal amplification‐based method was used for staining multiple targets in paraffin‐embedding specimens with the Opal 4‐Color Manual IHC Kit (NEL810001KT; Akoya Biosciences). Slides were processed for antigen retrieval by a standard inverter microwave heating technique with either diluted 50X Envision FLEX Target Retrieval Buffer (pH 9.0, Dako, K8004) for CD68 and CD86 for 15 min. Endogenous peroxidase activities were quenched using 3% hydrogen peroxide for 10 min at room temperature. Specimens were incubated with primary antibodies (CD68, 1:500, Cell Signaling Technology, CST97778S; CD86: 1:500, Cell Signaling Technology, CST19589S) overnight. The sections were then washed thoroughly and incubated with Opal polymer HRP Mouse+Rabbit (Akoya Biosciences, ARH1001EA) for 30 min at room temperature. Followed by a brief wash with TBST, Opal fluorophore (1:100) was applied for CD68 (Opal 570), and CD86 (Opal 520) for 15 min at room temperature. The section slides were counterstained with DAPI solution (1:1000) and mounted for examination using a fluorescence microscope.

### 5.11 Statistical Analysis

All statistical analyses were performed using GraphPad Prism 9.0 software. Data are presented as mean ± standard deviation (SD). Student's *t*‐test was used for comparisons between two groups, and one‐way ANOVA with Tukey's post hoc test was used for comparisons among multiple groups. A *p*‐value less than 0.05 was considered statistically significant.

## Author Contributions

B.T., C.S., K.M.L., D.L., M. K. H. L., and J.C.H.C. performed chemical synthesis, antibody conjugation, and characterization. J.L. performed computational molecular docking studies and conducted in vitro assays. B.T., W.H.L., C.S., and C.S.W.Y. contributed to in vivo experiments and data interpretation. C.M.C. was responsible for laboratory resources and supervision. X.L. supervised peptide design and bioconjugation strategy and supervision. W.T.W. and L.M.C.C. provided resources and supervision, and T.K.W.L. provided overall project supervision and critical revisions and supervision. C.T.T.W. designed the experiment, coordinated the research, analyzed data, provided financial support, and wrote the manuscript with input from all authors. All authors have read and approved the final manuscript.

## Funding

This work was supported by the General Research Fund from the Hong Kong Research Grants Council (Grant Number: 15303321) and the Research Impact Fund (Grant Number: R5008‐22). We also acknowledge support from the Innovation and Technology Commission, Innovation and Technology Fund—Innovation and Technology Support Programme (Project Number: ITS/013/22FP).

## Ethics Statement

All animal experiments were conducted in accordance with the institutional guidelines and approved by the Animal Ethics Committee of the Hong Kong Polytechnic University (Approval No. 20–21/154‐ABCT‐R‐GRF).

## Conflicts of Interest

The authors declare no conflicts of interest.

## Supporting information




**Supporting File S1**: mco270808‐sup‐0001‐SupMat.docx

## Data Availability

The data supporting the findings of this study are available within the article and its Supporting Information files.
