# Supplementary material for: Chemical‐Assembled Macrophage‐Engaging Glypican‐3 × Signal Regulatory Protein‐α (SIRP‐α) Bispecific Antibody for Immunotherapy Against Liver Cancer
Source: MedComm (2020). 2026 Jun 17;7(7):e70808. doi: 10.1002/mco2.70808 (PMC13273848; doi:10.1002/mco2.70808)
Supplement: Supplementary file 1 — Supporting File S1: mco270808‐sup‐0001‐SupMat.docx [file MCO2-7-e70808-s001.docx]

*Supporting Information*

Chemical-Assembled Macrophage-Engaging Glypican-3 × Signal Regulatory Protein-α (SIRP-α) Bispecific Antibody for Immunotherapy Against Liver Cancer

Bo Tang^a^, Wing Hei Leung^a^, Chihao Shao^a^, Dongfang Li^b^, Kwai Man Lau^a^, Marco K. H. Lui^a^, Jingyi Liu^a^, Jacky C. H. Chu^b^, Clare S. W. Yan^a^, Chi-Ming Che^b^, Xuechen Li^b^, Wing-Tak Wong^a^, Larry Ming Cheung Chow^a^, Terence K. W. Lee^a^, and Clarence T. T. Wong^a,c^

**Affiliations:**

^a^ Department of Applied Biology and Chemical Technology, The Hong Kong Polytechnic University, Hung Hom, Hong Kong SAR, China

^b^ Department of Chemistry, University of Hong Kong, Pokfulam, Hong Kong SAR, China

^c^ PolyU-BGI Joint Research Centre for Genomics and Synthetic Biology in Global Ocean Resources, The Hong Kong Polytechnic University, Hung Hom, Hong Kong SAR, China

**Contents**

**Experimental Section**

**Scheme S1**. The synthetic scheme of dendritic bispecific antibody conjugated with **A**. dimeric glypican-3-binding peptide and **B**. monomeric glypican-3-binding peptide.

**Figure S1**. Reverse-phase HPLC chromatogram and MALDI-TOF mass spectrum of glypican-3-binding peptide.

**Figure S2**. Reverse-phase HPLC chromatogram and MALDI-TOF mass spectrum of glypican-3-binding peptide dimer and glypican-3-binding peptide monomer.

**Figure S3**. Reducing SDS-PAGE of the conjugation of non-specific human IgG with different equivalents of peptide dimers.

**Figure S4**. Mass spectrometry analysis of the antibody-to-peptide dimer ratio of dBsAb generated by reacting anti-SIRP-α monoclonal antibody with 5, 10, 25, and 50 equivalents of peptide dimers.

**Figure S5**. ELISA binding experiment against glypican-3 and SIRP-α recombinant protein using different dBsAbs produced by different reaction conditions with the molar ratio of antibody to peptide dimer at 1:5, 1:10, 1:25, and 1:50.

**Figure S6**. Competitive ELISA demonstrating glypican-3 protein binding of monomeric peptide bispecific antibody (mdBsAb) and dBsAb with or without monomeric peptide blocking.

**Figure S7**. Molecular Docking of glypican-3 peptide monomer and dimer against glypican-3 protein.

**Figure S8**. 2D molecular docking projection interaction between glypican-3-binding peptide monomer and dimer against glypican-3 protein.

**Figure S9**. Flow cytometry results of cellular binding experiment of 50 nM dBsAb against HEK293, MHCC97L, and RAW264.7 for 1 hour at 4˚C.

**Figure S10**. Serum stability of dBsAb was assessed using an ELISA binding assay.

**Figure S11**. MTT cell viability assay of glypican-3 peptide dimer against different cell lines.

**Figure S12**. Fluorescent microscopy of macrophage-cancer cell adhesion experiment in the presence and absence of dBsAb, mdBsAb, or mAb.

**Figure S13**. Competitive ELISA of SIRP-α-CD47 blockage by dBsAb.

**Figure S14.** Confocal images and zoomed-in view of antibody-mediated cellular phagocytosis of macrophages against A549, MHCC97L, and HepG2 cells in the presence of dBsAb or mAb (50 nM) for 12 hours.

**Figure S15.** Quantification of macrophage phagocytosis by flow cytometry.

**Figure S16**. Flow cytometry plots and bar graph data show phagocytic activity of macrophages against MHCC97L and HepG2 cells treated with PBS, mAb, mdBsAb, or dBsAb.

**Figure S17**. RT-qPCR experiment on investigating the antigen presentation process.

**Figure S18**. Images of xenograft biopsies collected from mice after treatment with dBsAb and mAb, compared to control PBS treatment.

**Figure S19**. Multiplex immunofluorescent staining of CD68^+^ macrophage in the mice tumor sections under different treatments.

**Experimental Section**

**General**

*N*,*N*-Dimethylformamide (DMF) and dichloromethane (CH₂Cl₂) were dried over molecular sieves prior to use. Acetonitrile was of HPLC grade, while all other solvents were of analytical grade and used without further purification. Reverse-phase HPLC separations were carried out using an XTerra^TM^ MS C18 column (5 µm, 3.9 mm × 150 mm) at a flow rate of 0.4 mL/min for analytical purposes, or an NUCLEODUR C18 HTec column (5 µm, 21 mm × 250 mm) at 7 mL/min for preparative purposes. The system used included a Waters 1525 binary pump and a Waters 2998 photodiode array detector. The gradient for analysis was set as follows: solvent A was 0.1% trifluoroacetic acid (TFA) in acetonitrile, and solvent B was 0.1% TFA in deionized water. The gradient started at 5% A and 95% B for the first 5 min, changed to 15% A and 85% B over 10 min, then transitioned to 100% A over 30 min, maintained for 5 min, returned to 0% A and 100% B for 5 min, and held for an additional 5 min. Electrospray ionization (ESI) mass spectra were recorded on an Agilent 6546 LC/Q-TOF mass spectrometer. Antibody analysis was performed using a MAbPac Reversed Phase HPLC Column (4 μm, 3 mm × 50 mm) at a flow rate of 0.3 mL/min. The conditions for analysis were as follows: solvent A was 0.1% formic acid (FA) in deionized water, and solvent B was 0.1% FA in acetonitrile. The gradient started at 80% A and 20% B for the first 0.5 min, then changed to 40% A and 60% B over 3 min, held for 5 min, and reverted to 80% A and 20% B over 0.5 min, maintained for 1 minute. Size-exclusion chromatography was carried out on a Zenix-C SEC-300 Gel Filtration column (3 μm, 7.8 mm × 300 mm) at a flow rate of 1 mL/min using an Agilent 1260 Infinity II LC system with an Agilent 1260 Quat Pump and an Agilent 1260 DAD WR detector. The mobile phase consisted of sodium phosphate (150 mM, pH 7.0), used isocratically for 10 min.

**Molecular Dynamic (MD) simulation**

MD simulations were conducted to evaluate the structural stability of glypican-3 binding peptides, monomer and dimer, in complex with the target protein. The initial complex structures were obtained from molecular docking using AutoDock Vina previously, and subsequently subjected to two independent MD simulations. Each for a duration of 100 ns under constant temperature (300 K) and pressure (1 atm). All simulations were performed using GROMACS version 2025.4 with the Amber98 force field1-3. Missing loop regions in the protein were modeled using Modeller. Each system was solvated in a cubic water box, and the ionic strength was adjusted to physiological saline concentration. System electroneutrality was maintained by adding Na+ and Cl- counterions4, 5. Final RMSD trajectories (.xvg files) were processed and visualized using custom scripts using Python 3.13 to generate publication-quality plots.

**Preparation of glypican-3-binding peptide**

The peptide AcNH-CALLANHEELFQT-COHN_2_ was synthesized using a CEM Liberty Blue Automated Microwave Peptide Synthesizer with a modified 9-fluorenylmethoxycarbonyl (Fmoc) solid-phase peptide synthesis protocol, employing commercially available *N*-α-Fmoc-protected amino acids. Rink amide resin served as the solid support. The Fmoc protecting group was removed using 20% piperidine in DMF, and *N*,*N*’-diisopropylcarbodiimide (DIC) was used as the carboxyl group-activating agent. Each coupling reaction was performed at room temperature using excess Fmoc-protected amino acid (4 equiv.), DIC (4 equiv.), and ethyl cyano(hydroxyimino)acetate (Oxyma) (8 equiv.) in DMF. N-terminal acetylation was carried out by stirring the peptide with CH_2_Cl_2_/acetic anhydride/*N*,*N*-diisopropylethylamine (DIPEA) (1:1:0.1 v/v/v) at room temperature for 30 min. Following washing, the peptide was cleaved and deprotected with a solution of 95% TFA, 2.5% triisopropylsilane (TIS), and 2.5% deionized water for 1 hour. The resin was filtered out, and the filtrate was precipitated by adding to the cold diethyl ether. After centrifugation and removal of the supernatant, the precipitated peptide was lyophilized to obtain the crude product, which was subsequently purified by reverse-phase HPLC and lyophilized again.

**Preparation of glypican-3 × SIRP-α dBsAb and mdBsAb**


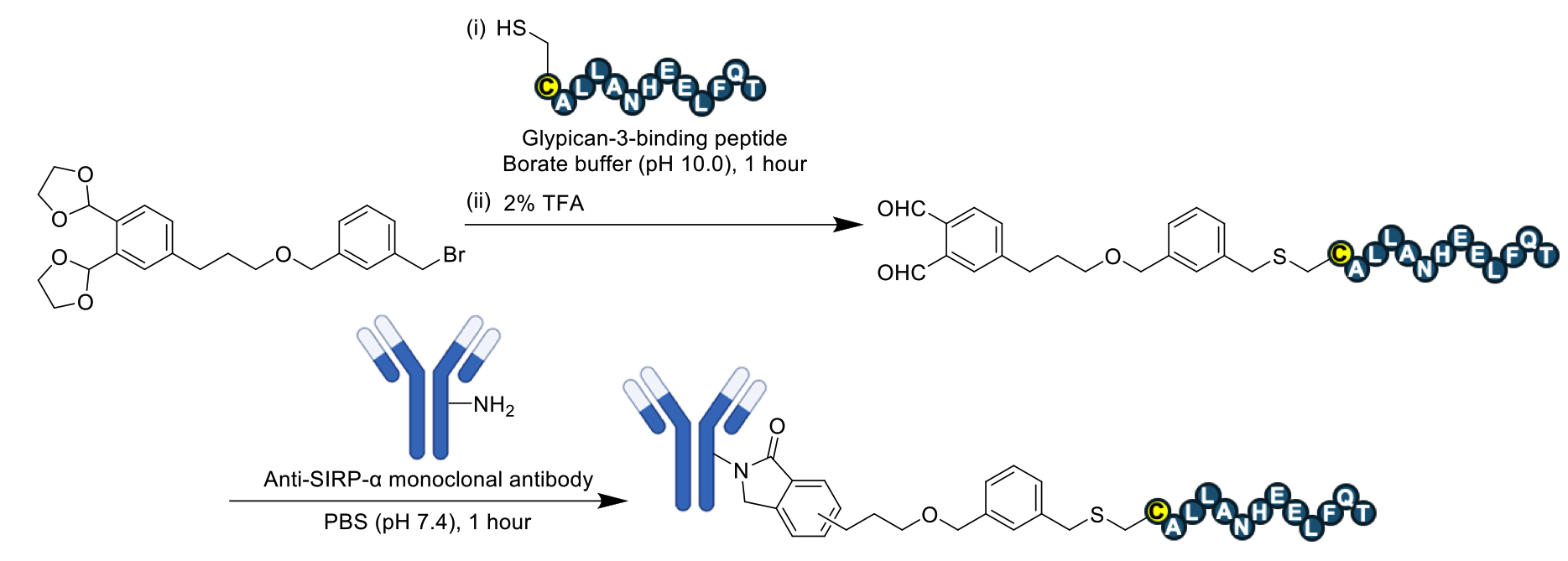


**Scheme S1**. The synthetic scheme of dendritic bispecific antibody conjugated with **A**. dimeric glypican-3-binding peptide and **B**. monomeric glypican-3-binding peptide.

The bifunctional linker (2.0 mM) was dissolved in borate buffer (50 mM, pH 10.0) and reacted with the glypican-3-binding peptide (4.0 mM) for 1 hour at room temperature to form the peptide dimer (A) or monomer (B). The reaction mixture was then treated with 2% TFA to remove the ortho-phthalaldehyde (OPA) protecting group, followed by neutralization with 1 M NaOH to pH 7.4. The rabbit anti-SIRP-α monoclonal antibody (Sino Biological, China, Cat. No. 50956-R001) (0.2 mg/mL) was diluted in PBS (pH 7.4) to a final concentration of 0.02 mg/mL. Without further purification, the peptide dimer solution was added to the antibody solution at various molar ratios (antibody:dimer = 1:5, 1:10, 1:25, 1:50, and 1:100) and reacted for 1 hour at room temperature. The conjugation processes were monitored by ESI-MS analysis. The reaction mixture was filtered through a Zeba Spin Desalting Column (40K MWCO) by centrifugation at 1500 g for 2 min to remove excess unconjugated peptide dimer, yielding the purified glypican-3 × SIRP-α dBsAb. The final product was lyophilized and stored as a powder at -20°C until further use.

**Cell lines and culture conditions**

MHCC97L human hepatocellular carcinoma cells (from Liver Cancer Institute, Fudan University, China), HepG2 human liver cancer cells (ATCC no. HB-8065), HEK293 human embryonic kidney cells (ATCC no. CRL-1573), A549 human lung carcinoma cells (ATCC no. CCL-185), and RAW264.7 murine macrophage cells were maintained in Dulbecco's Modified Eagle Medium (DMEM) (ThermoFisher Scientific, cat. no. 12100-046) supplemented with fetal bovine serum (FBS) (10%) and penicillin‐streptomycin (100 unit mL^-1^ and 100 μg mL^-1^, respectively). All the cells were grown at 37 ˚C in a humidified 5% CO_2_ atmosphere.

**Serum stability assay**

A solution of 50% human serum (Sigma-Aldrich, USA) in PBS was prepared. Subsequently, dBsAb or mAb at 50 nM were added to the serum-PBS solution and incubated at 37°C. At different time intervals (0-48 hours), 100 µL of solution was aliquoted from the samples and followed by rapid flash-freezing in liquid nitrogen to arrest any biochemical reactions. The samples were later thawed and analyzed by ELISA to determine the binding affinity to target antigens.

**MTT cell viability assay**

Approximately 5 × 10^3^ HepG2, A549, HEK293, MHCC97L cells per well were seeded in a 96-well cell culture plate and cultured at 37°C overnight. A ten-fold serial dilution of the glypican-3 binding peptide dimer, starting at 10 µM, was added to the plate and incubated for 24 hours at 37°C. After 24-hour incubation, the cells were washed three times with PBS. A 100 μL volume of 0.5 mg mL-1 3-(4,5-dimethylthiazol-2-yl)-2,5-diphenyltetrazolium bromide (MTT) reagent was added to each well, and the plates were incubated for 4 h at 37°C. The MTT solution was replaced with 50 μL DMSO for 10 min to solubilize the purple formazan crystals formed. Absorbance was read at 492 nm using the Thermo Scientific Varioskan LUX Multimode Microplate Reader.

**RT-qPCR for antigen presentation activity**

1 × 10^5^ Raw 264.7 cells and 1 × 10^5^ HepG2 or A549 cancer cells were seeded in a 12-well cell culture plate alongside dBsAb or mAb (50 nM) overnight at 37°C. Total RNA was extracted using the total RNA isolation reagent (Biosharp) according to the manufacturer’s guidelines and reverse-transcribed via reverse-transcription kit (Biosharp). For real-time quantitative PCR (RT-qPCR), cDNA was performed using SYBR Green qPCR mix (Biosharp) using specific primers. RT-qPCR was performed on a QuantStudio 5 Real-Time PCR System (Applied Biosystems) and analyzed using QuantStudio™ Design & Analysis Software. All gene expression results were normalized to the geometric mean of 18S housekeeping genes for each condition. Gene expressions were analyzed by technical duplicates using at least three independent biological replicates.

Primers for mouse 18S

Forward (5’ 🡪 3’): GTAACCCGTTGAACCCCATT

Reverse (5’ 🡪 3’): CCATCCAATCGGTAGTAGCG

Primers for mouse H2-Eb1

Forward (5’ 🡪 3’): GCGGAGAGTTGAGCCTACG

Reverse (5’ 🡪 3’): ACCATCTGACTTCAATGTTGCC

**Pharmacokinetic and organ toxicity studies**

The pharmacokinetic profile of the dBsAb was determined by using female BALB/c mice (9–11 weeks old), and they were administered a single intravenous dose of 3 mg/kg dBsAb via the tail vein. At predetermined time points (10, 60, 240, 1440, 4320, and 7200 min after injection), groups of mice (n = 3 per time point) were euthanized by ketamine and xylazine, followed by cardiac puncture for whole blood collection. Serum was isolated by centrifugation at 4000 × g for 15 min at 4°C, followed by rapid flash-freezing in liquid nitrogen, and stored at –80°C until analysis. The sample was diluted 5000-fold, and the concentration of dBsAb in mouse serum was quantified by a sandwich ELISA. dBsAb concentrations were determined by interpolation from a standard curve generated using serial dilutions of purified dBsAb.


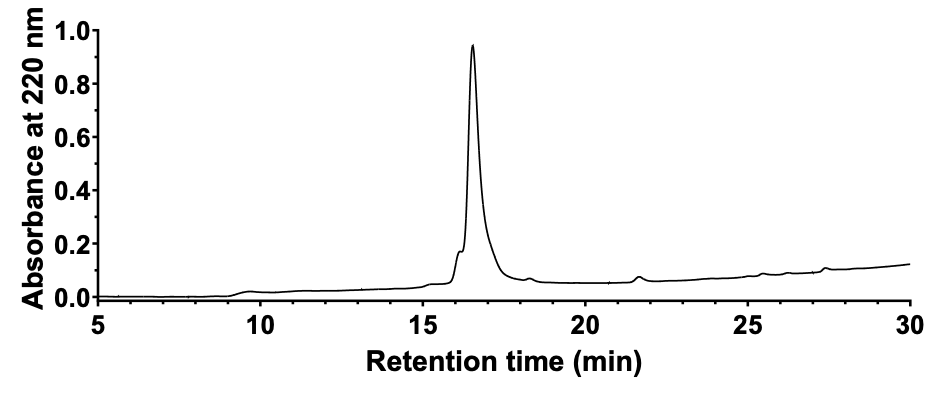


A.

B.


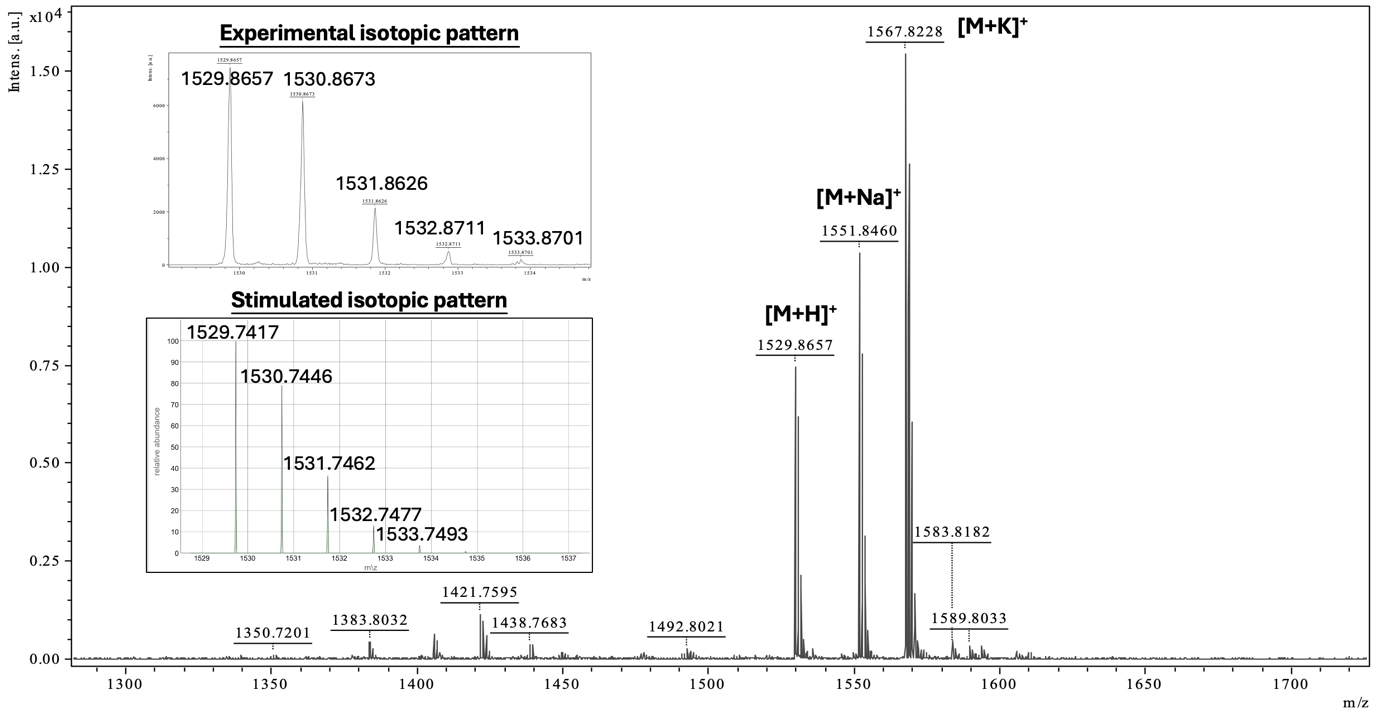


**Figure S1.** Reverse phase-HPLC chromatogram (A) and MALDI-TOF mass spectrum (B) of glypican-3-binding peptide: HRMS (MALDI-TOF): *m/z* calcd for C_67_H_105_N_18_O_21_S^+^ [M+H]^+^, 1529.7417; found, 1529.8657, [M+Na]^+^, 1551.8460, [M+K]^+^, 1567.8228.


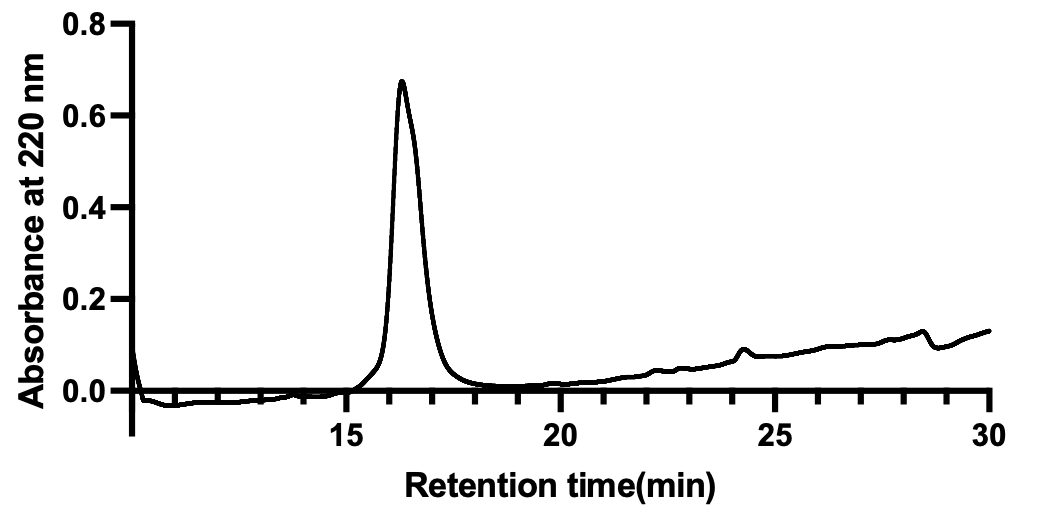


A.

B.


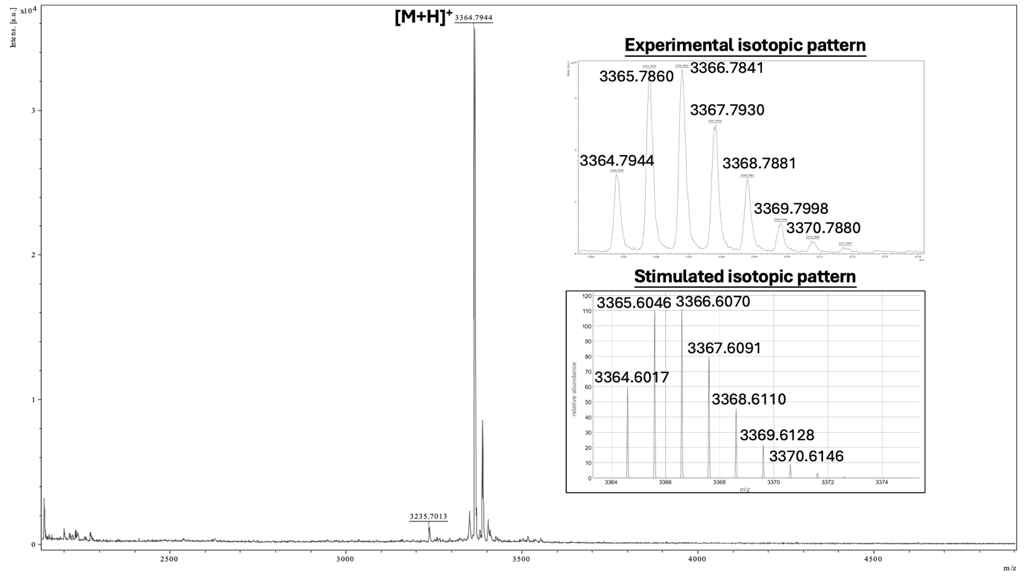


C.


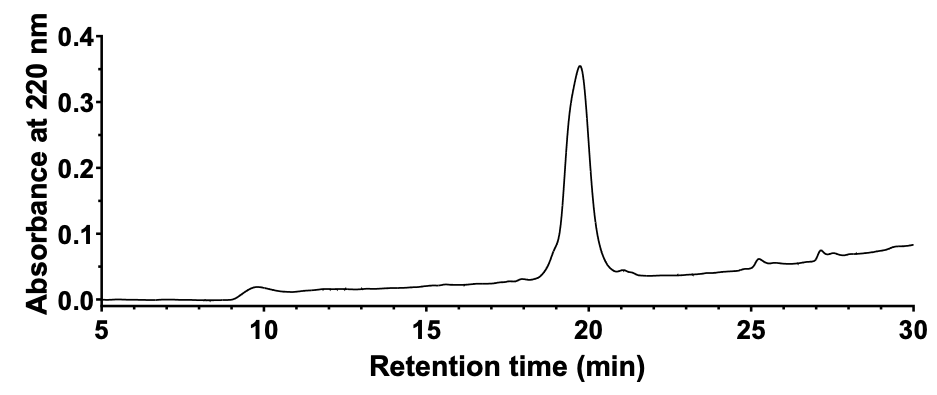


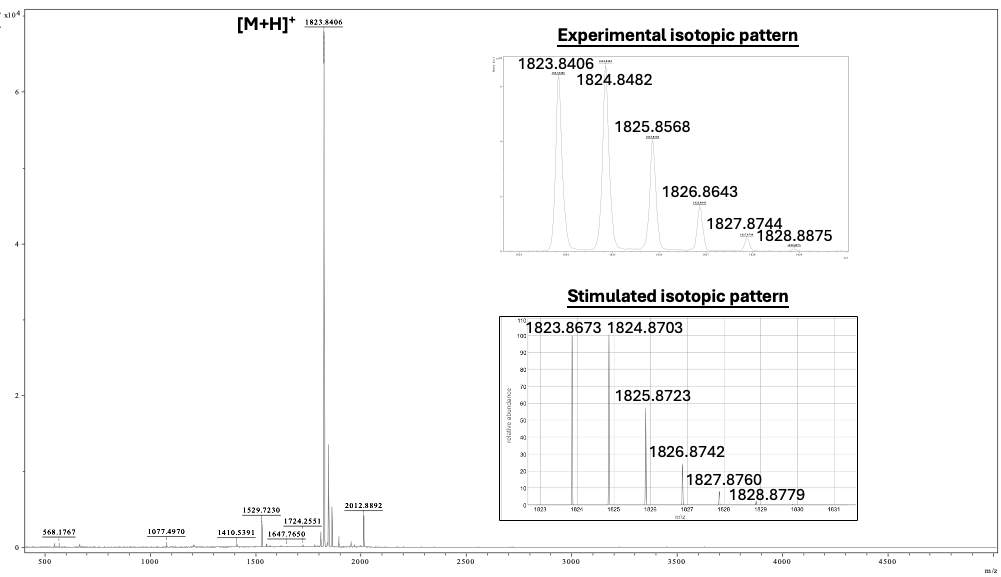


D.

**Figure S2.** Reverse phase-HPLC chromatogram (A) and MALDI-TOF mass spectrum (B) of glypican-3-binding peptide dimer: HRMS (MALDI-TOF): *m/z* calcd for C_154_H_227_N_36_O_45_S_2_^+^ [M+H]^+^, 3364.6017; found, 3364.7944. Reverse phase-HPLC chromatogram (C) and MALDI-TOF mass spectrum (D) of glypican-3-binding peptide monomer: HRMS (MALDI-TOF): *m/z* calcd for C_86_H_123_N_18_O_24_S^+^ [M+H]^+^, 1823.8673; found, 1823.8406.


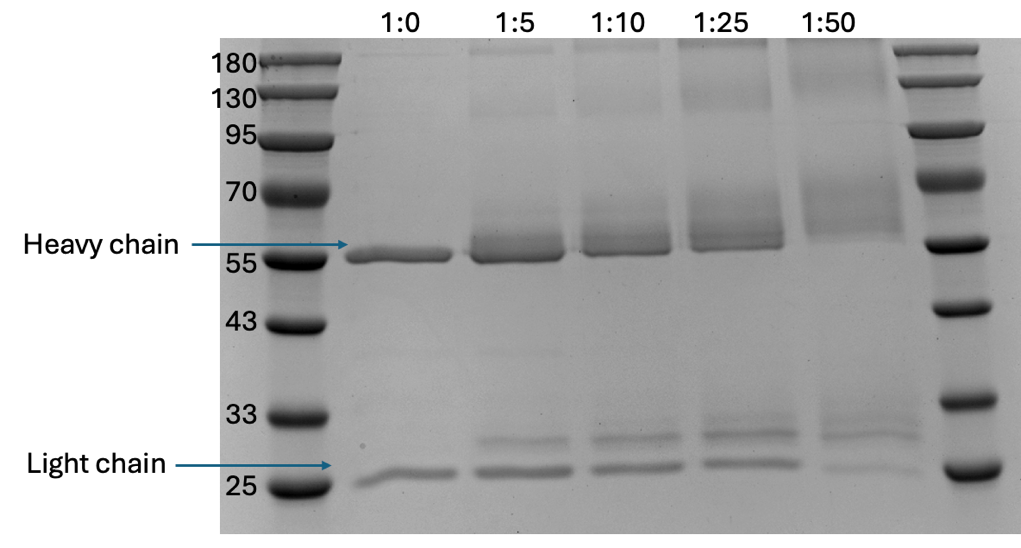


**Figure S3.** Reducing SDS-PAGE analysis showing the conjugation of non-specific human IgG with varying amounts of peptide dimer. The SDS-PAGE results demonstrate that increasing the peptide dimer ratio caused an upward shift in both the light and heavy chains. The appearance of smeared bands indicates that peptides were conjugated at different positions on the antibody, resulting in a heterogeneous mixture of products with varying numbers of conjugated peptides.


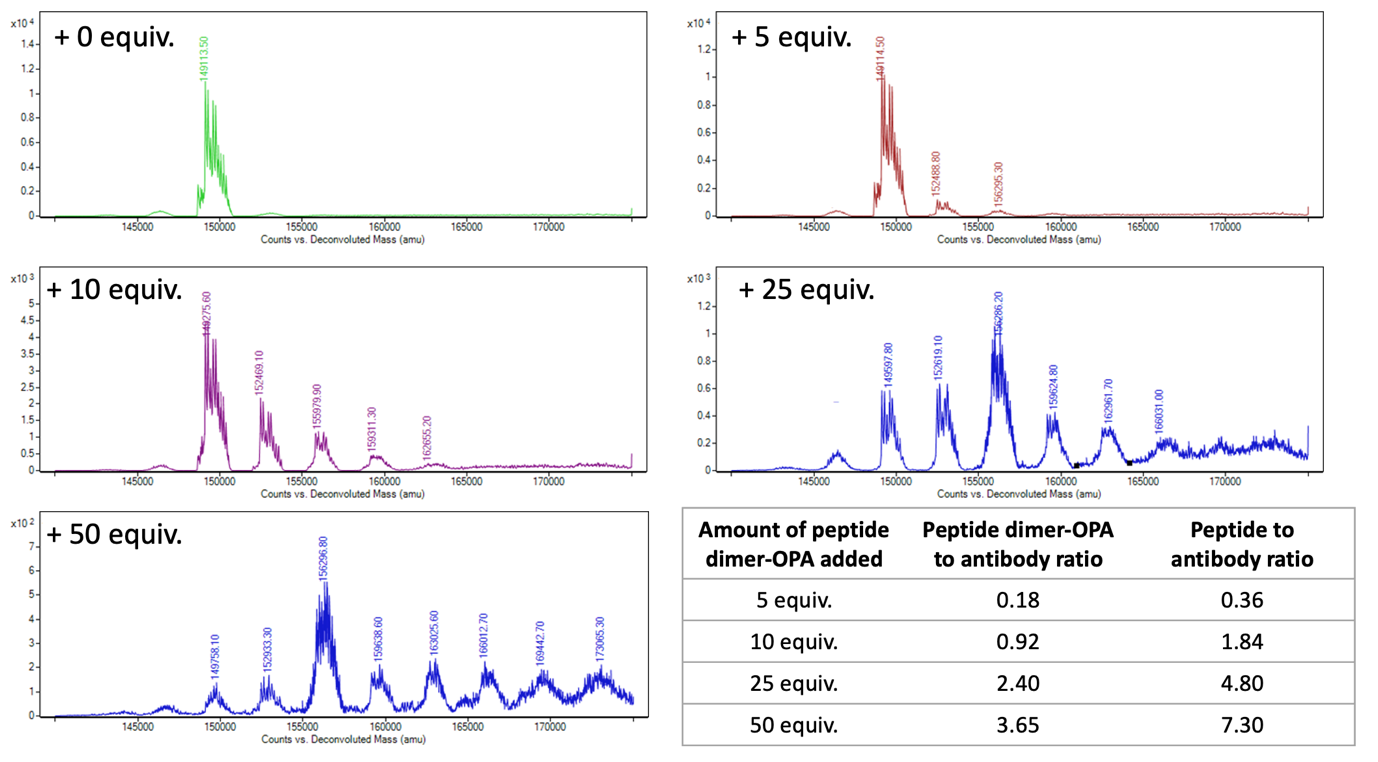


**Figure S4**. Mass spectrometry analysis of the antibody-to-peptide dimer ratio of dBsAb generated by reacting anti-SIRP-α monoclonal antibody with 5, 10, 25, and 50 equivalents of peptide dimers.


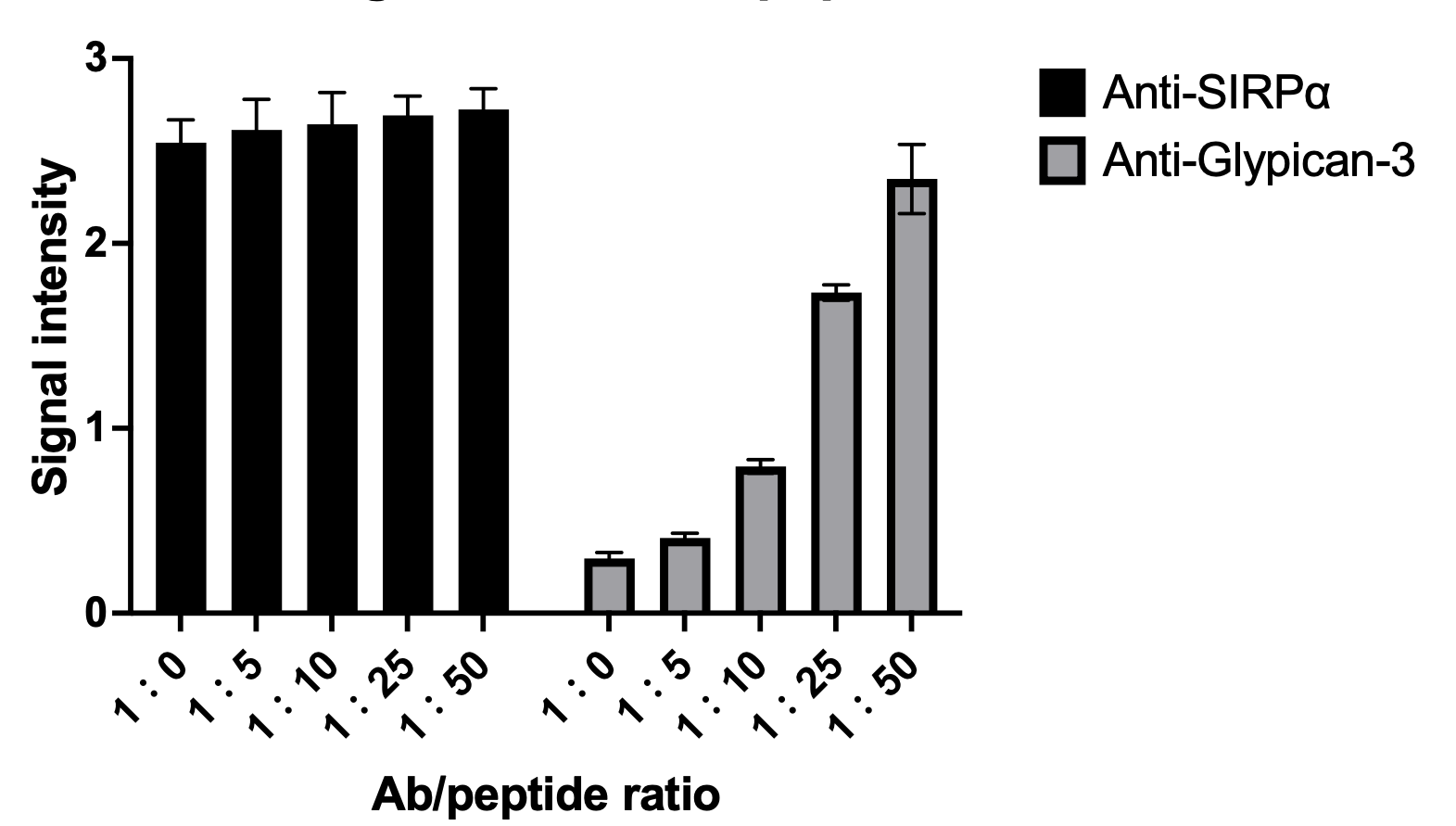


**Figure S5**. ELISA binding experiment against glypican-3 and SIRP-α recombinant protein using different dBsAbs produced by different reaction conditions with the molar ratio of antibody to peptide dimer at 1:0,1:5, 1:10, 1:25, and 1:50.


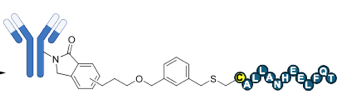

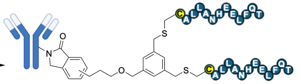

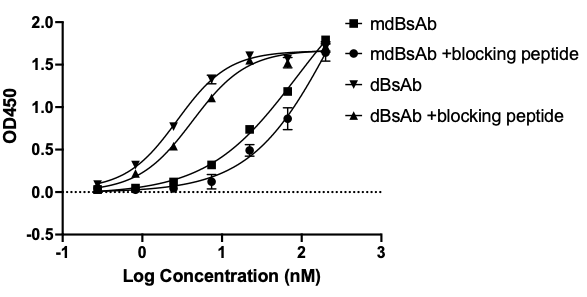


**Figure S6**. Competitive ELISA demonstrating glypican-3 protein binding of monomeric peptide bispecific antibody (mdBsAb) and dBsAb with or without monomeric peptide blocking. The EC50 values for mdBsAb, mdBsAb with blocking peptide, dBsAb, dBsAB with blocking peptide are 86.42 nM, 248.1 nM, 2.752 nM, and 4.273 nM respectively. The glypican-3 binding of mdBsAb is significantly reduced upon blocking with 0.1 mM monomeric peptide (AcNH-ALLANHEELFQT-CONH_2_) in 2% BSA in PBS with 0.05% Tween-20, suggesting epitope overlap. In contrast, the high glypican-3 binding of dBsAb retains despite blocking, indicating enhanced avidity and rebinding kinetics. The data suggest that both formats bind the same site, but dimerization improves effective binding strength due to multivalent interactions.


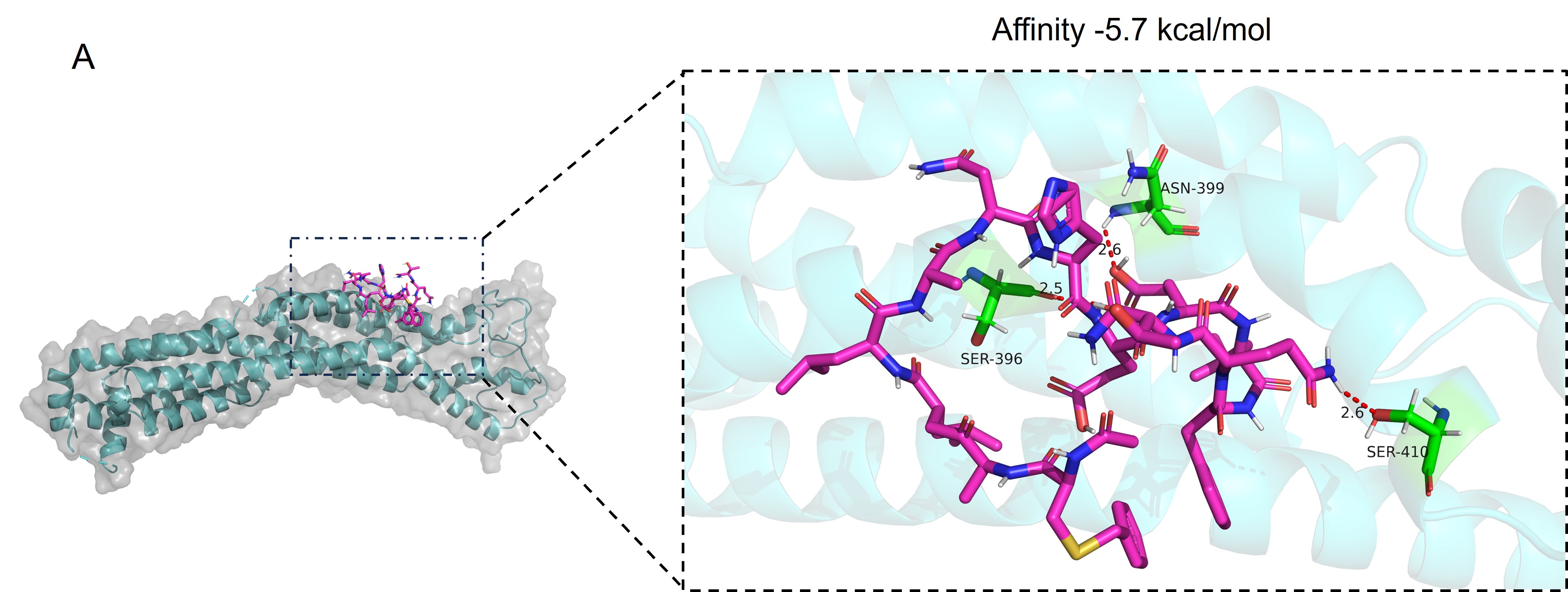

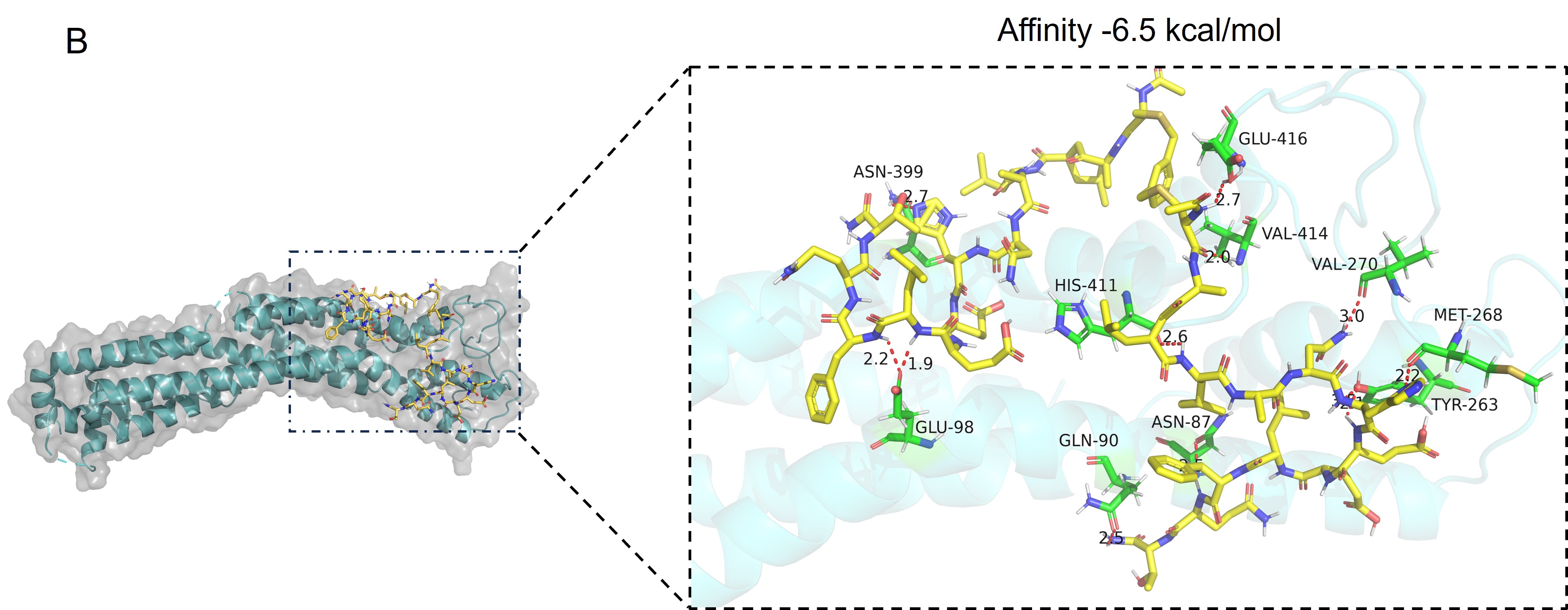


**Figure S7**. (A) Binding affinity and docking poses of glypican-3-binding peptide monomer (magenta) to glypican-3 receptor(cyan). The residues (green) from glypican-3 receptor are labelled. (B) Binding affinity and docking poses after docking of glypican-3-binding peptide dimer (yellow) and glypican-3 receptor(cyan). The residues (green) from glypican-3 receptor are labelled.


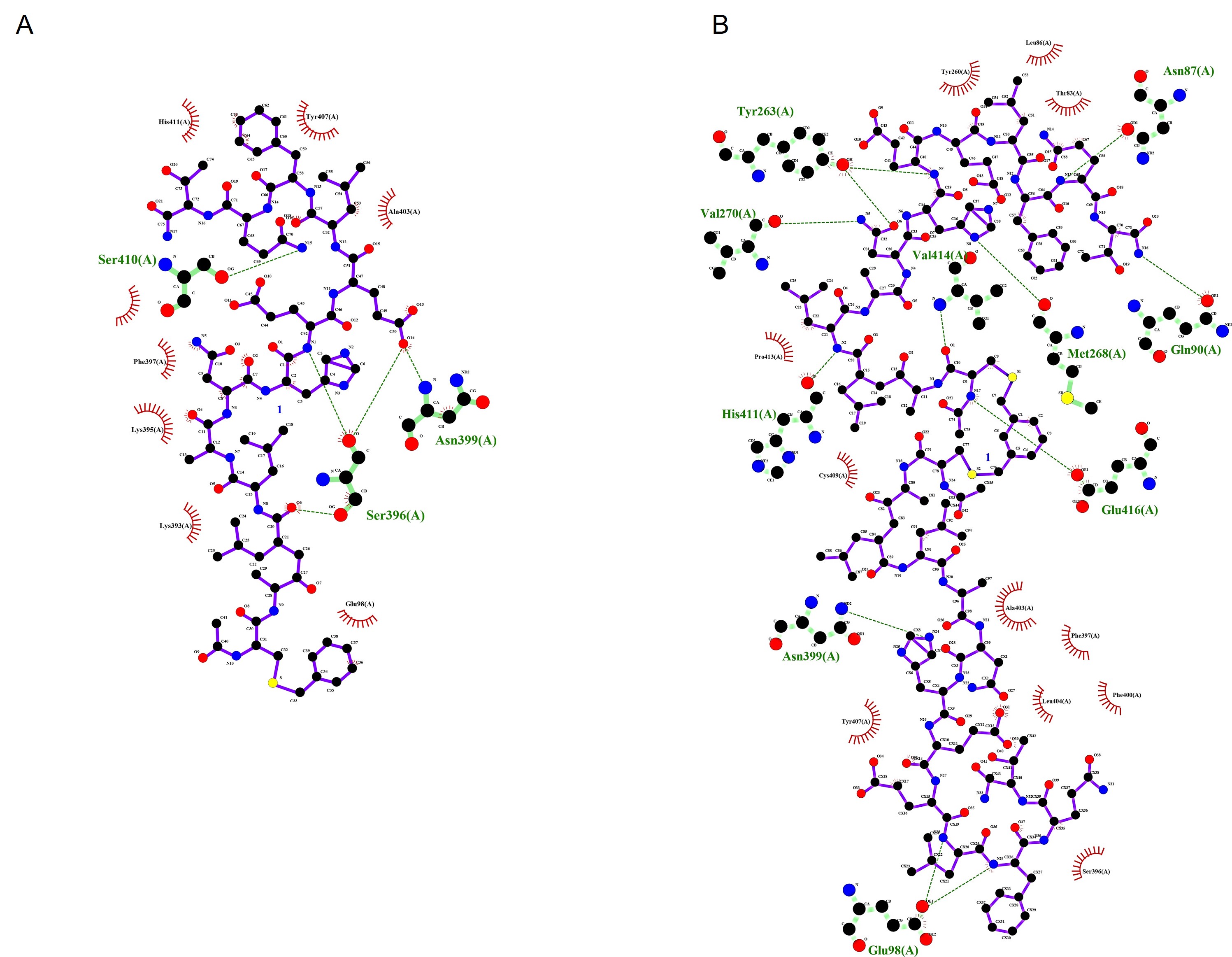


**Figure S8**. 2D molecular docking projection interaction between glypican-3-binding peptide monomer and dimer against glypican-3 protein. The carbon, nitrogen, oxygen, and sulphur atoms are presented as balls in black, blue, red, and yellow balls, respectively. The polar interactions are shown in dark green dashed lines. The residues in glypican-3 that form polar interaction with ligands are labelled in green. The ligands are shown in purple stick. The residues are shown in light green stick. (A) Glypican-3 protein: glypican-3-binding peptide monomer. (B) Glypican-3 protein: glypican-3-binding peptide dimer.


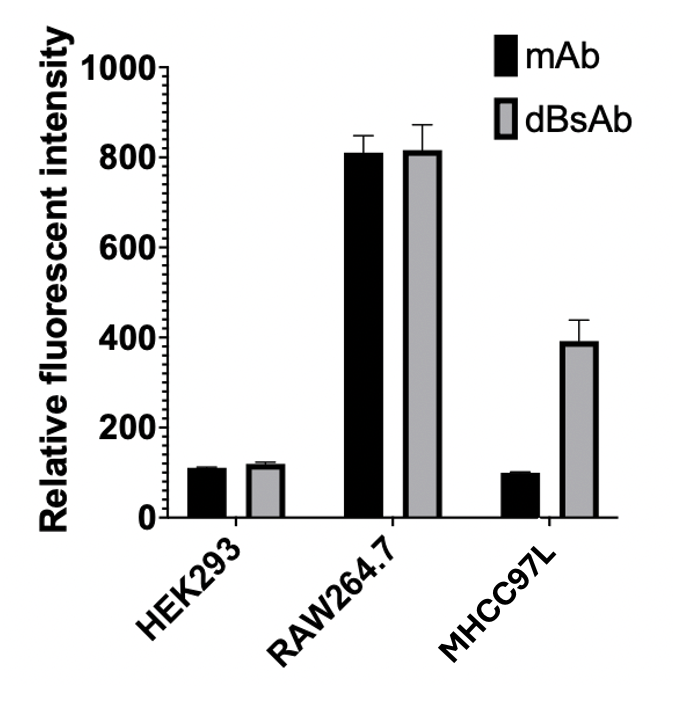


**Figure S9.** Flow cytometry results of cellular binding experiment binding of 50 nM dBsAb against HEK293, MHCC97L, and RAW264.7 for 1 hour at 4˚C for 1 hour. The data showed that the dBsAb is specific towards both macrophages and liver cancer cells.


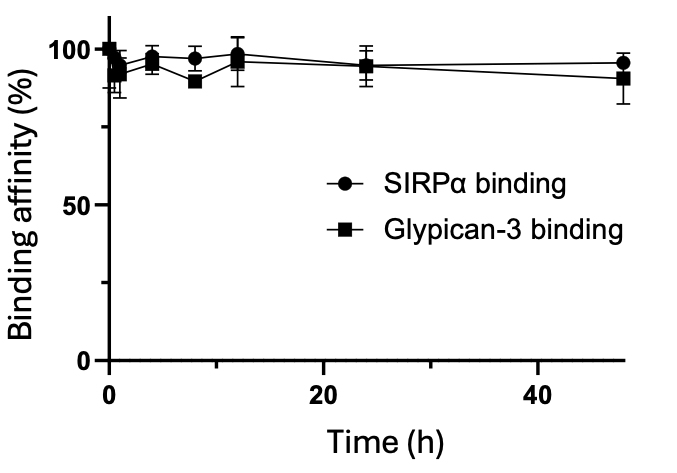


**Figure S10.** Serum stability of dBsAb was assessed using an ELISA binding assay. The percentage change in binding affinity of dBsAb to SIRP-α and glypican-3 was measured after incubation with 50% serum over 48 hours.


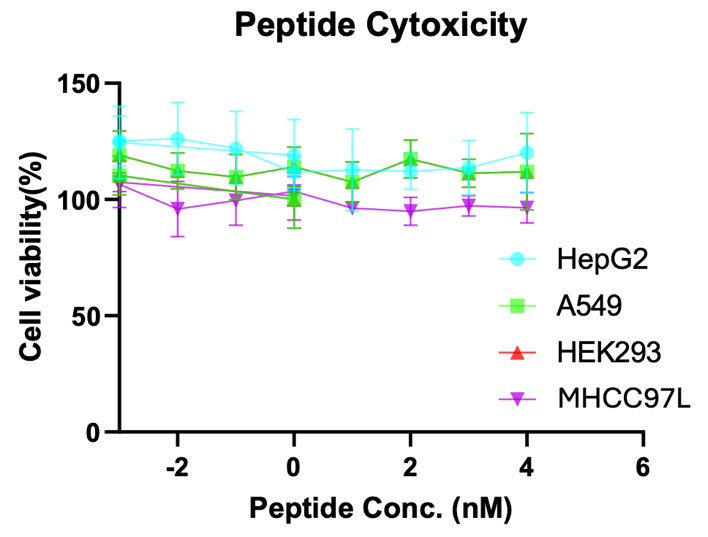


**Figure S11.** MTT cell viability assay of glypican-3 peptide dimer against different cell lines. The data showed that the peptide dimer has no significant cytotoxicity against the above cell lines.


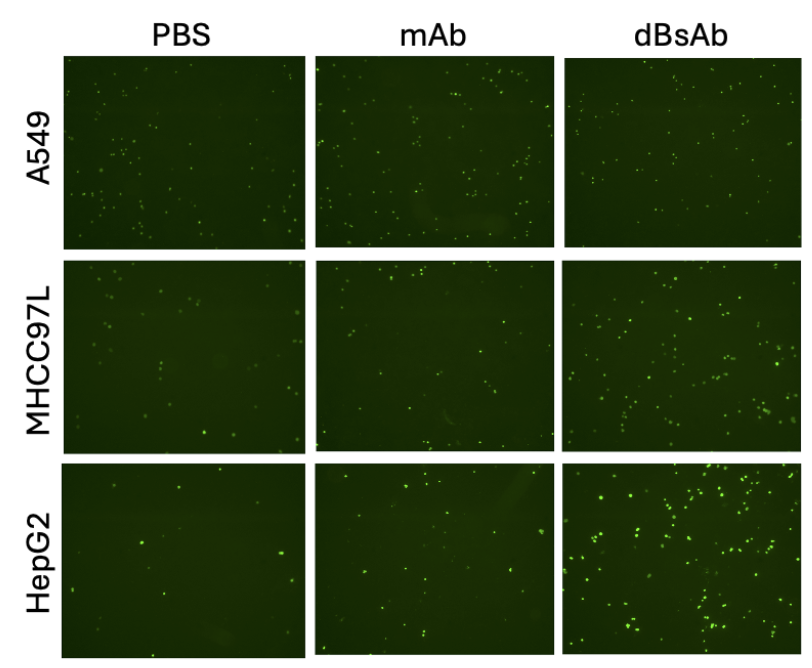

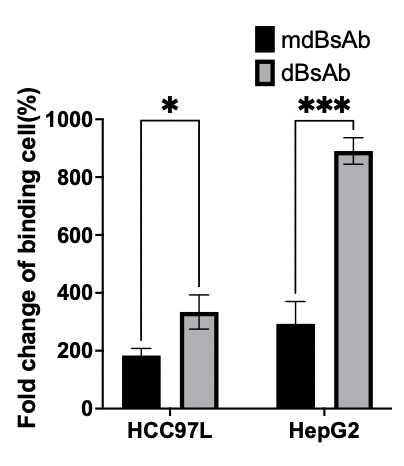


**Figure S12**. Fluorescent microscopy of cell-cell adhesion experiment in the presence and absence of dBsAb or mAb (Left). The data showed that dBsAb can enhance cell-cell interaction only in liver cancer cell lines HepG2 and MHCC97L. The fold change of binding cell was calculated by binding cell number of treatment/ binding cell number of PBS × 100%. Quantification of fold change in cell binding (right) showed dBsAb induced a markedly higher fold increase in macrophage-cancer cell adhesion than mdBsAb in both MHCC97L (**p* < 0.05) and HepG2 (***p* < 0.001).


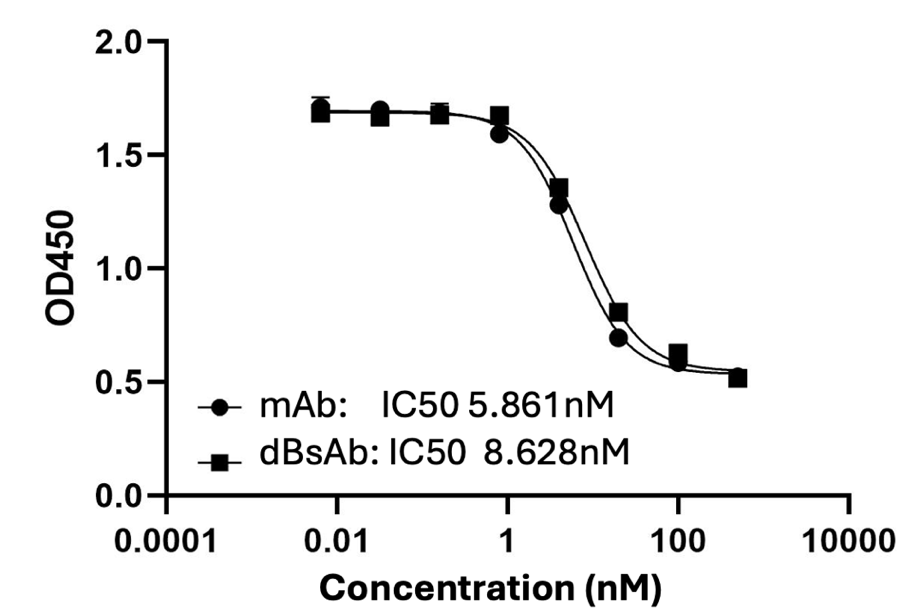


**Figure S13.** Competitive ELISA of SIRP-α-CD47 blockage by dBsAb or mAb. They both could effectively disrupted CD47-SIRP-α interaction. The data shown are mean ± SEM derived from three experiments, each conducted with three technical replicates.


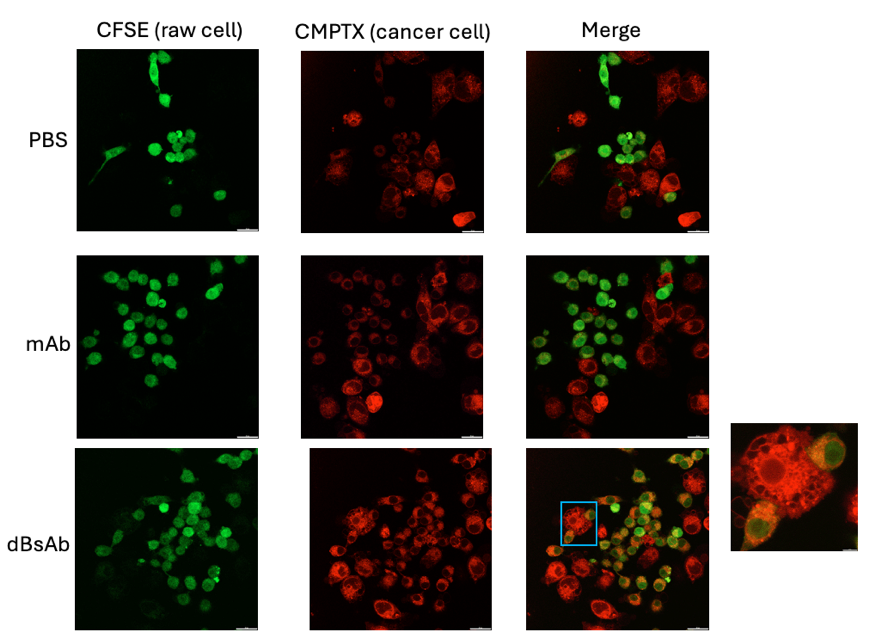

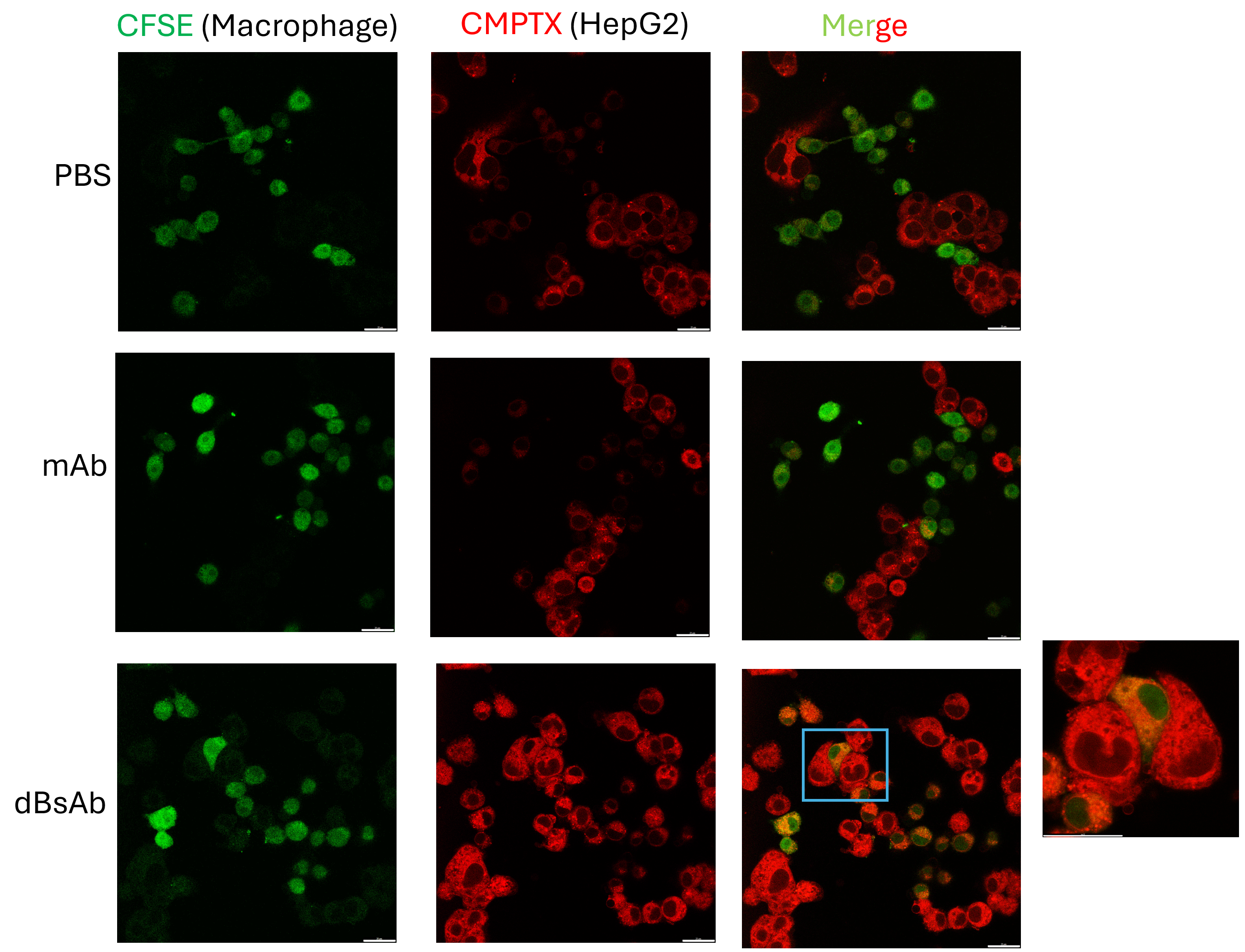


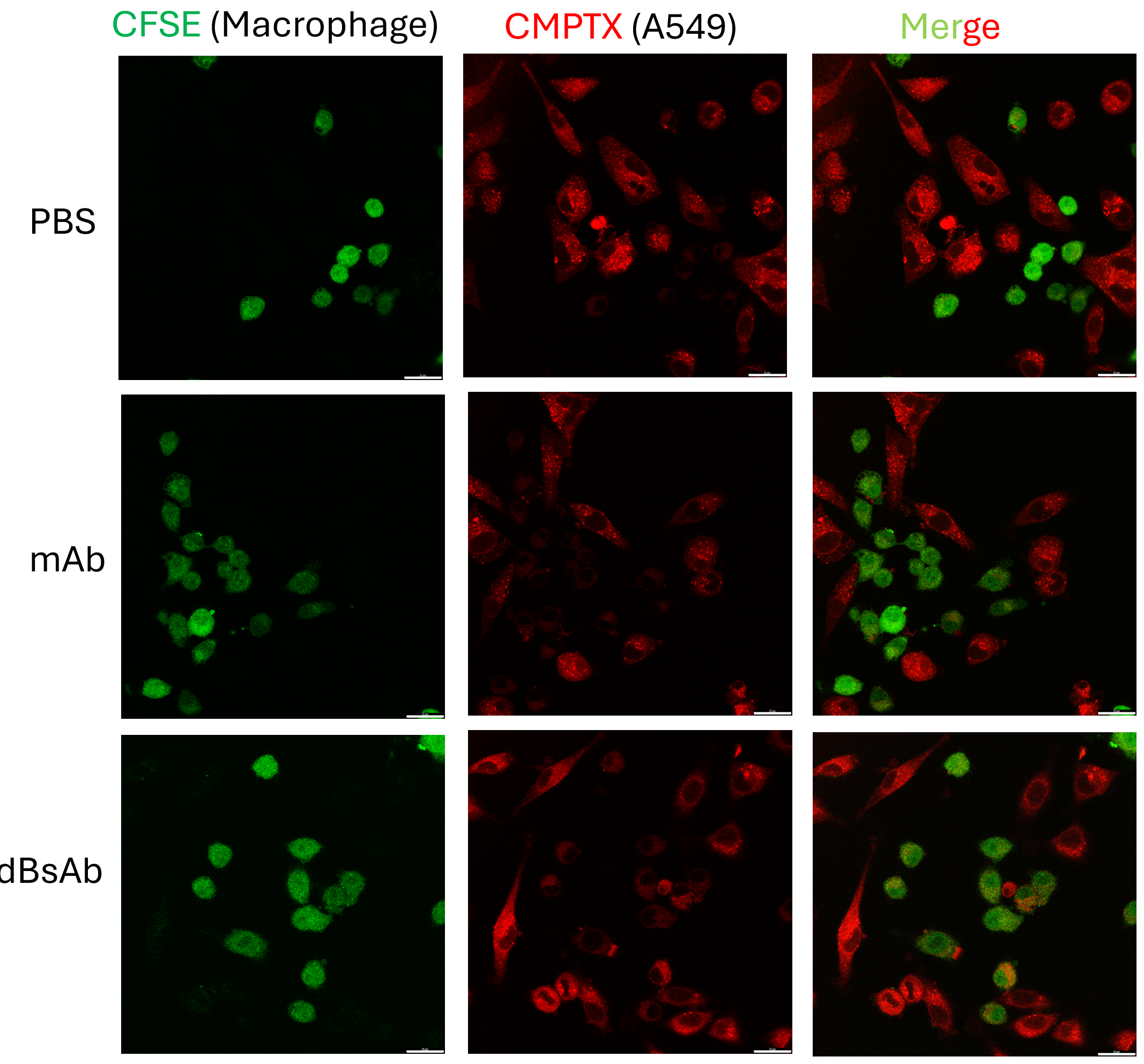


**Figure S14.** Confocal images and zoomed-in view of antibody-mediated cellular phagocytosis of macrophages (green) against A549, MHCC97L, and HepG2 cells (red) in the presence of dBsAb or mAb (50 nM) for 12 hours.


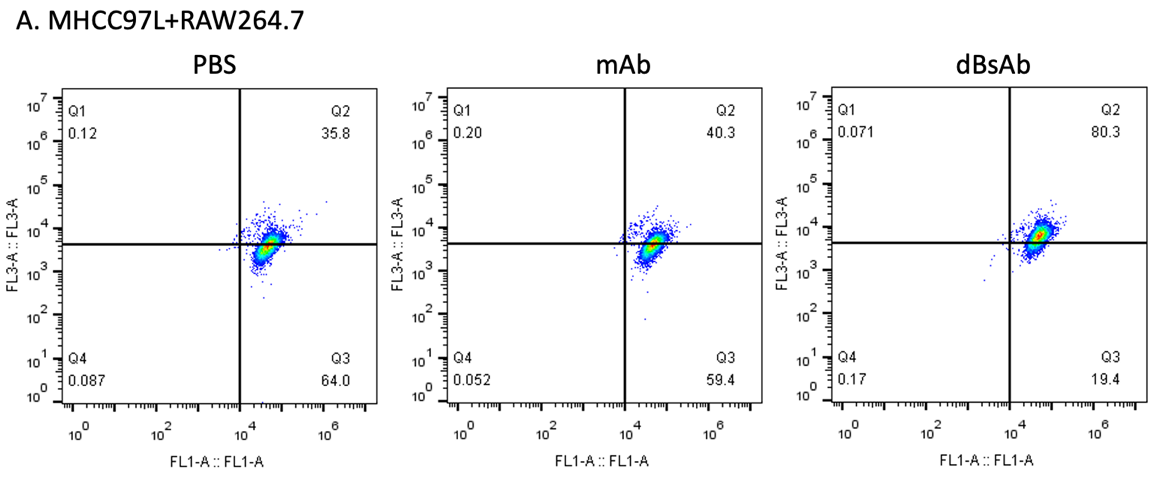

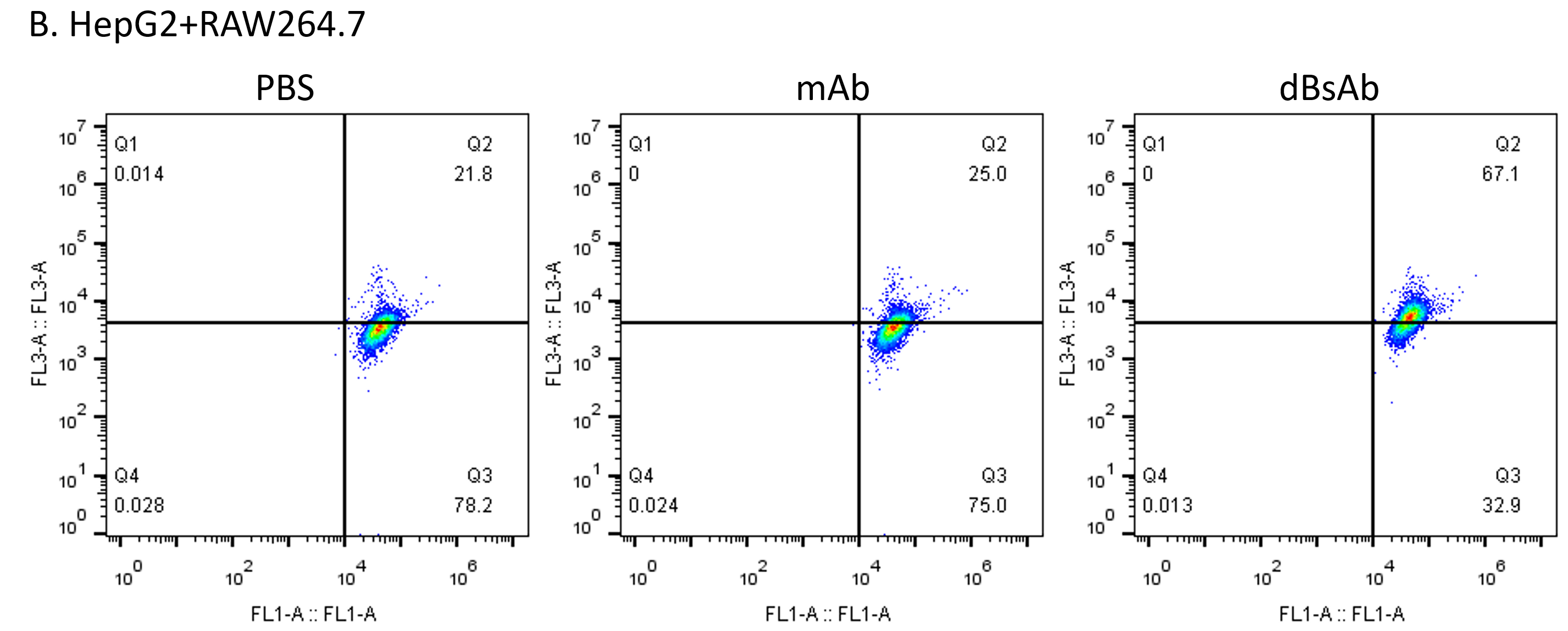

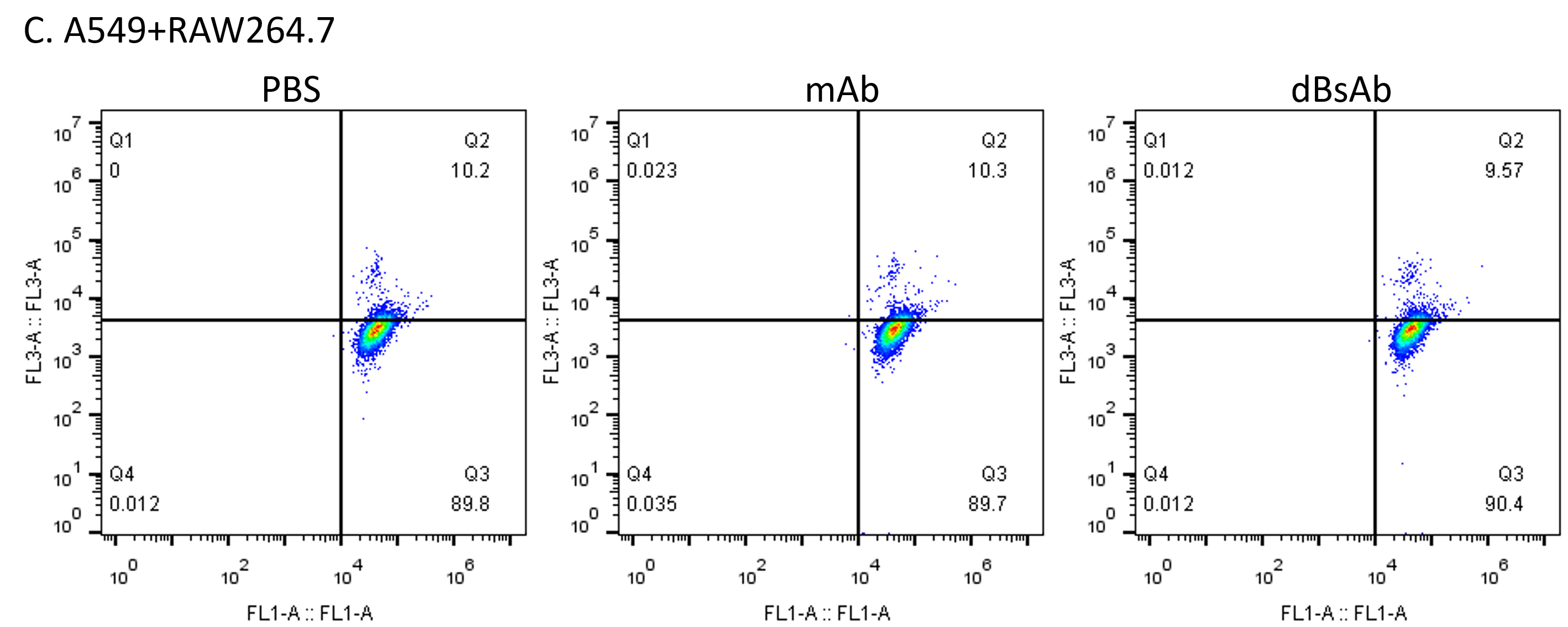

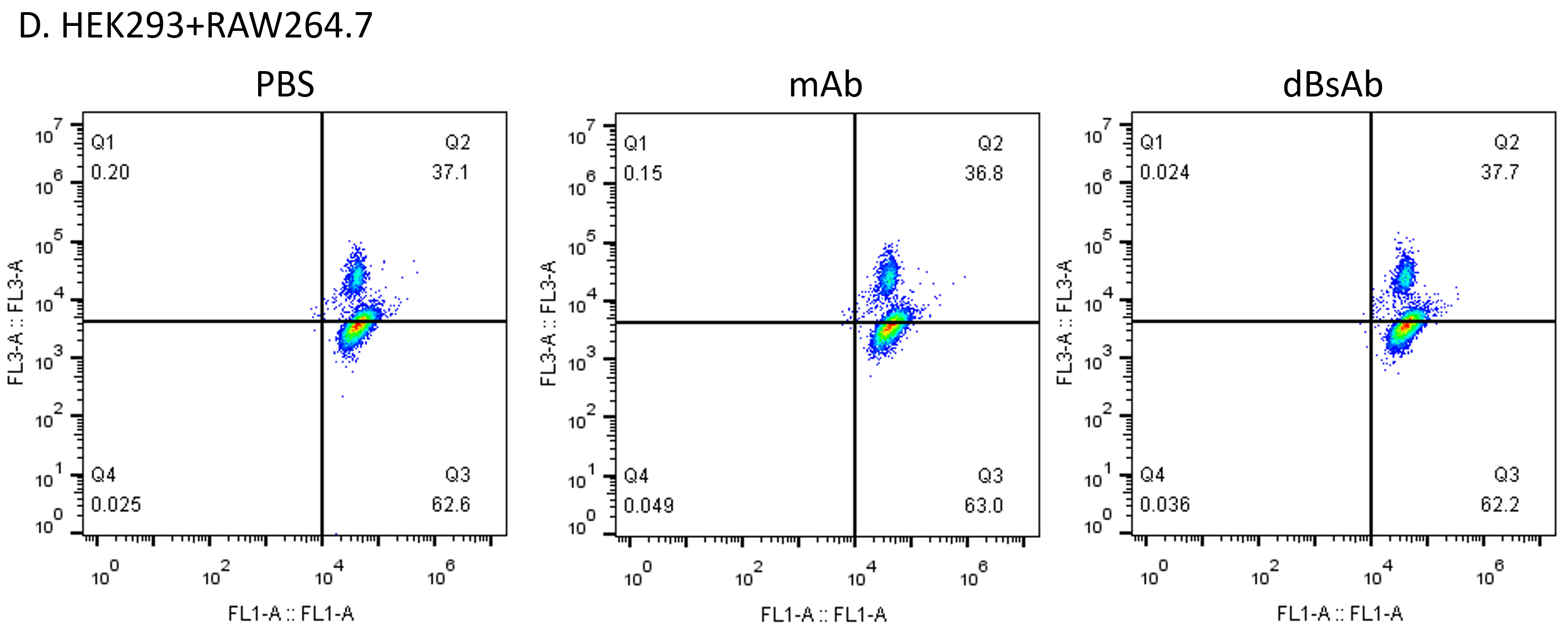


**Figure S15.** Quantification of macrophage phagocytosis by flow cytometry. dBsAb treatment specifically enhanced the phagocytic activity of macrophages against HepG2 and MHCC97L liver cancer cells, as evidenced by increased double-positive populations, while no significant difference was observed for the control HEK293 cell line.


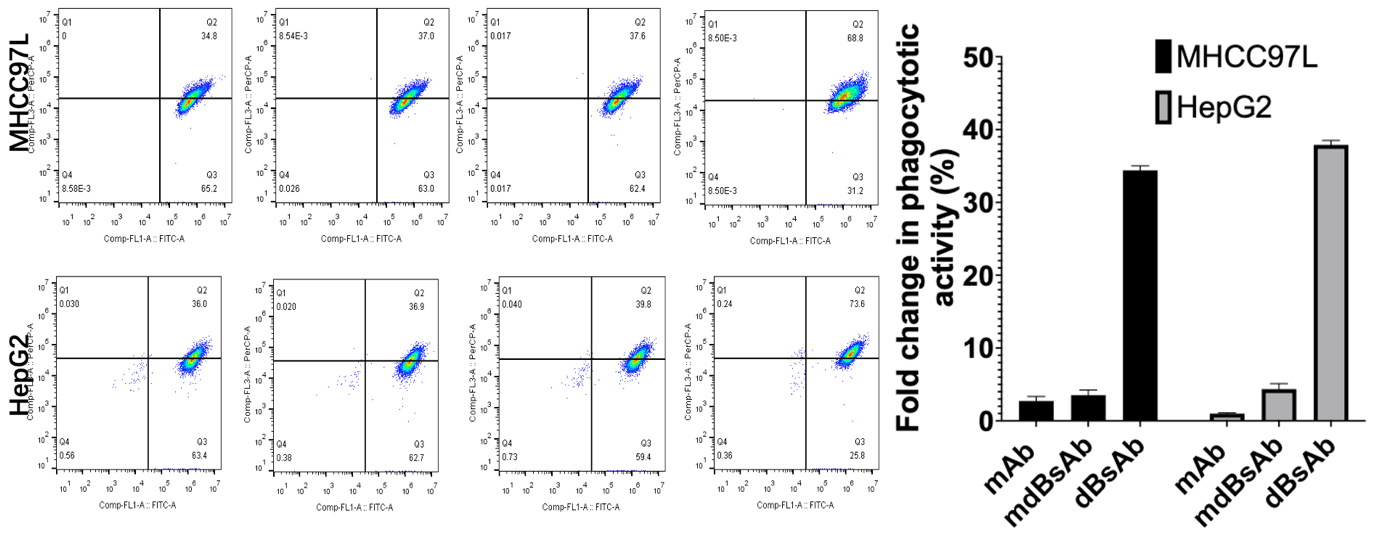
**Figure S16**. Flow cytometry plots and bar graph data show phagocytic activity of macrophages against MHCC97L and HepG2 cells treated with PBS, mAb, mdBsAb, or dBsAb.


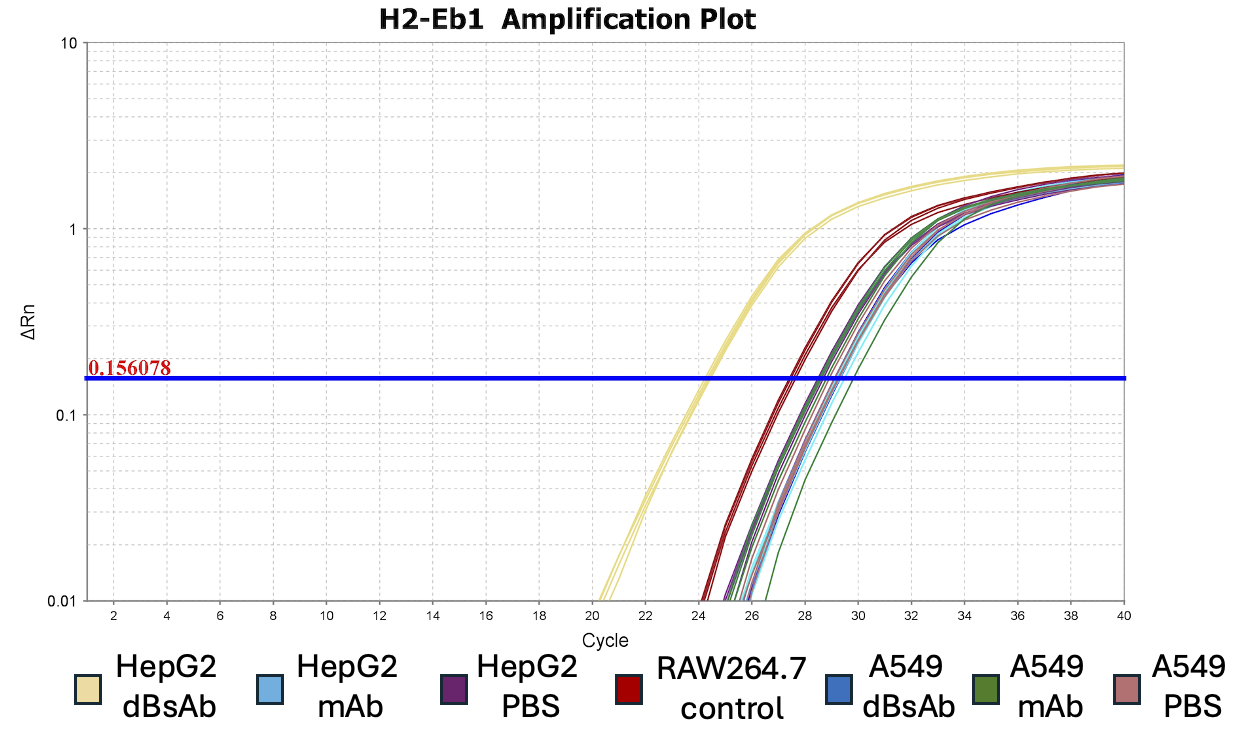
**Figure S17.** RT-PCR experiment on investigating the antigen presentation process. The RAW264.7 macrophage was incubated with different cell lines under different treatments. The expression of the H2-Eb1 gene, which encodes a key component of the major histocompatibility complex class II (MHC-II) involved in antigen presentation in macrophages, was evaluated in co-cultures with HepG2 cells treated with dBsAb. While other treatments against HepG2 cells or the treatments against the A549 cells did not show the upregulation of H2-Eb1 in macrophages, indicating that our novel dBsAb could enhance the antigen presentation process in macrophages when incubated with glypican-3 positive HepG2 cells.


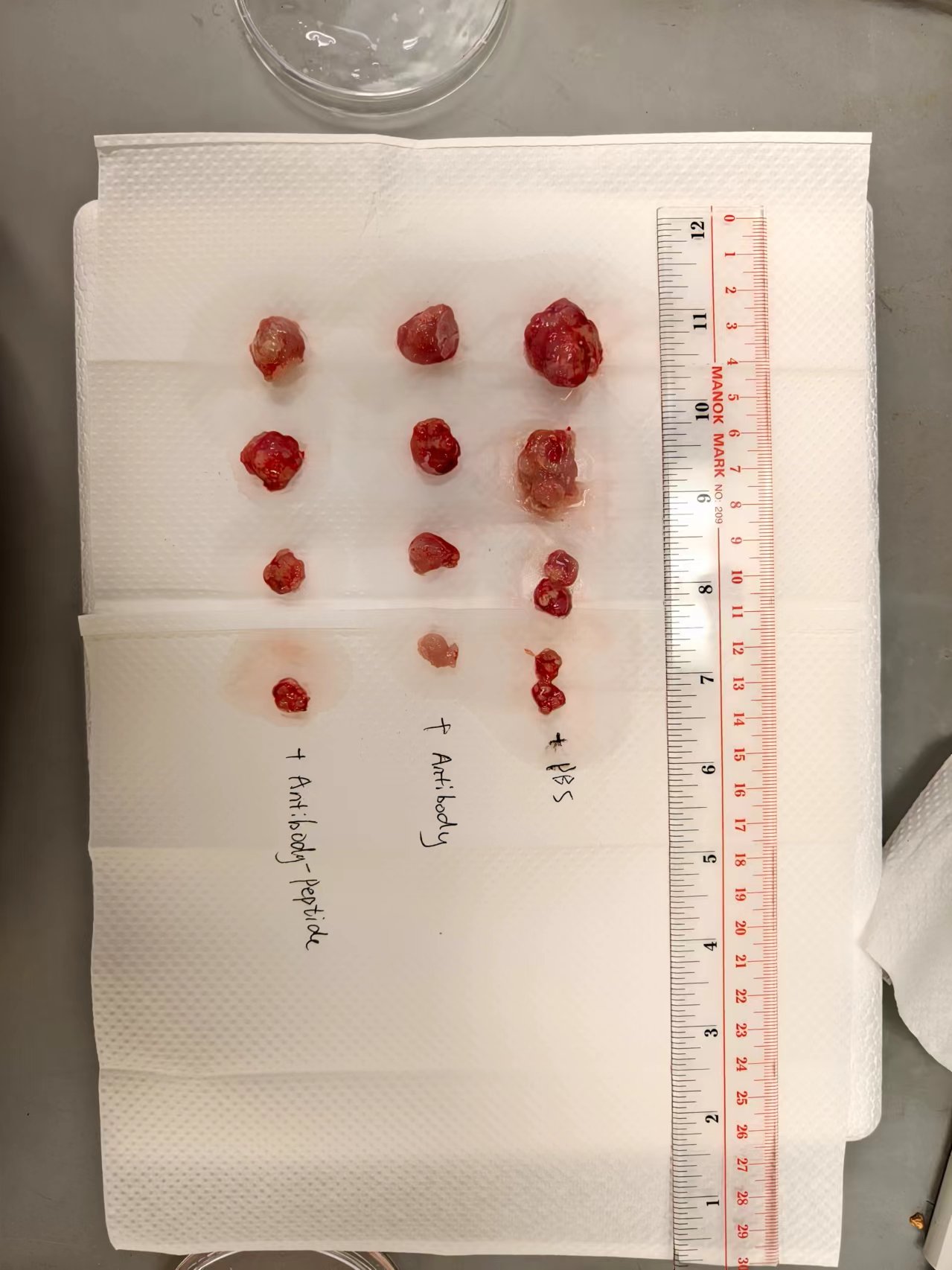


PBS

mAb

dBsAb

**Figure S18.** Images of xenograft biopsies collected from mice after treatment with dBsAb and mAb, compared to control PBS treatment.


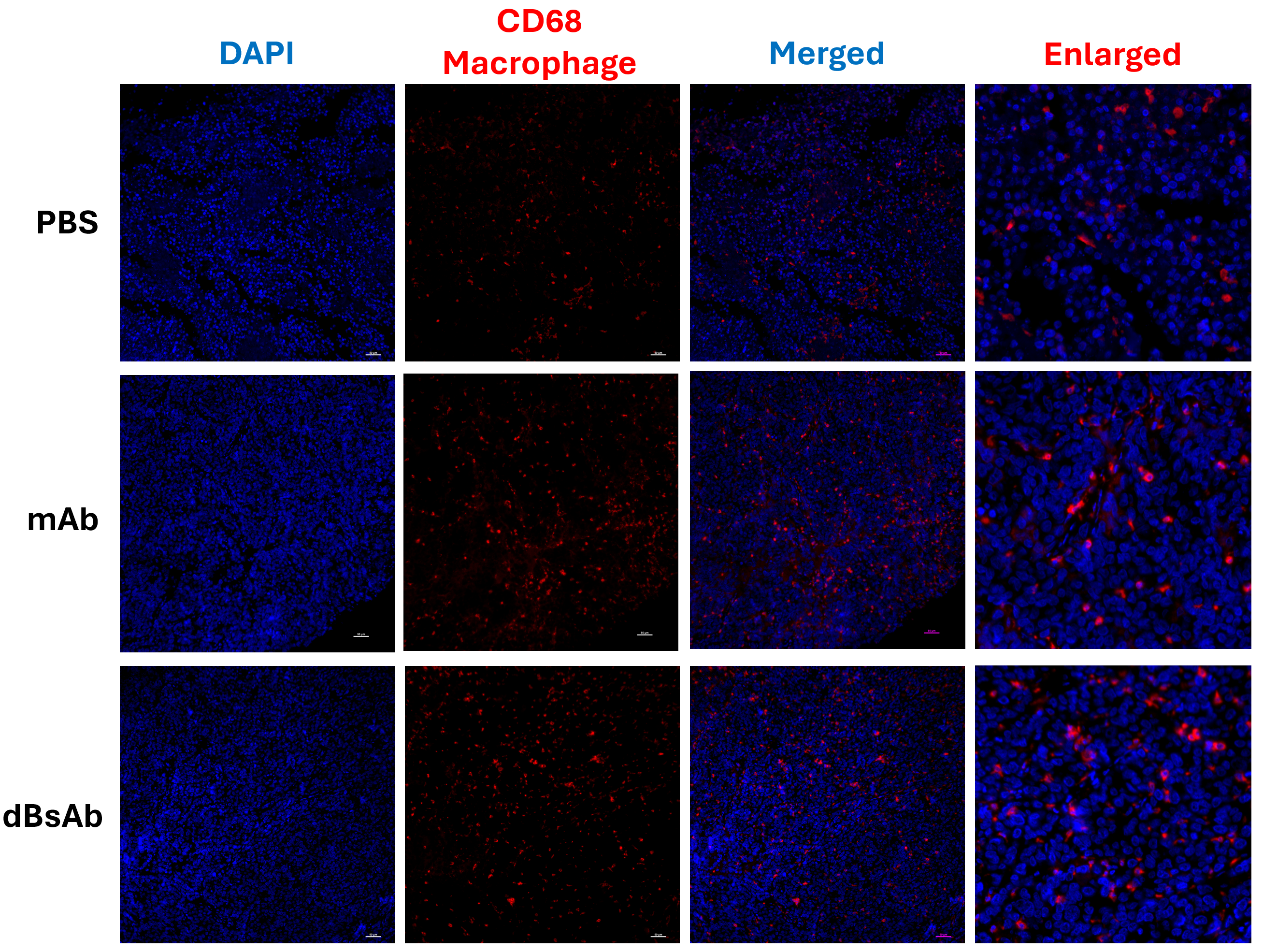


**Figure S19.** Multiplex immunofluorescent staining of CD68^+^ macrophage in the mice tumor sections under different treatments, including PBS, mAb, and dBsAb.

**References**

1. S. Páll, A. Zhmurov, P. Bauer, M. Abraham, M. Lundborg, A. Gray, B. Hess and E. Lindahl, *The Journal of chemical physics*, 2020, **153**.

2. M. J. Abraham, T. Murtola, R. Schulz, S. Páll, J. C. Smith, B. Hess and E. Lindahl, *SoftwareX*, 2015, **1**, 19-25.

3. J. Schulze, *Project report. Norwegian University of scince and technology*, 2017.

4. S. Jamal, A. Grover and S. Grover, *Frontiers in pharmacology*, 2019, **10**, 780.

5. D. Gheidari, M. Mehrdad and F. Hoseini, *Frontiers in Pharmacology*, 2024, **15**, 1360226.
